# Diversity of Pseudomonas Genomes, Including Populus-Associated Isolates, as Revealed by Comparative Genome Analysis

**DOI:** 10.1128/AEM.02612-15

**Published:** 2015-12-22

**Authors:** Se-Ran Jun, Trudy M. Wassenaar, Intawat Nookaew, Loren Hauser, Visanu Wanchai, Miriam Land, Collin M. Timm, Tse-Yuan S. Lu, Christopher W. Schadt, Mitchel J. Doktycz, Dale A. Pelletier, David W. Ussery

**Affiliations:** a Joint Institute for Computational Sciences, University of Tennessee, Knoxville, Tennessee, USA; b Molecular Microbiology and Genomics Consultants, Zotzenheim, Germany; c Biosciences Division, Oak Ridge Laboratory, Oak Ridge, Tennessee, USA

## Abstract

The Pseudomonas genus contains a metabolically versatile group of organisms that are known to occupy numerous ecological niches, including the rhizosphere and endosphere of many plants. Their diversity influences the phylogenetic diversity and heterogeneity of these communities. On the basis of average amino acid identity, comparative genome analysis of >1,000 Pseudomonas genomes, including 21 Pseudomonas strains isolated from the roots of native Populus deltoides (eastern cottonwood) trees resulted in consistent and robust genomic clusters with phylogenetic homogeneity. All Pseudomonas aeruginosa genomes clustered together, and these were clearly distinct from other Pseudomonas species groups on the basis of pangenome and core genome analyses. In contrast, the genomes of Pseudomonas fluorescens were organized into 20 distinct genomic clusters, representing enormous diversity and heterogeneity. Most of our 21 Populus-associated isolates formed three distinct subgroups within the major P. fluorescens group, supported by pathway profile analysis, while two isolates were more closely related to Pseudomonas chlororaphis and Pseudomonas putida. Genes specific to Populus-associated subgroups were identified. Genes specific to subgroup 1 include several sensory systems that act in two-component signal transduction, a TonB-dependent receptor, and a phosphorelay sensor. Genes specific to subgroup 2 contain hypothetical genes, and genes specific to subgroup 3 were annotated with hydrolase activity. This study justifies the need to sequence multiple isolates, especially from P. fluorescens, which displays the most genetic variation, in order to study functional capabilities from a pangenomic perspective. This information will prove useful when choosing Pseudomonas strains for use to promote growth and increase disease resistance in plants.

## INTRODUCTION

The genus Pseudomonas is one of the most diverse bacterial genera, currently containing >200 recognized species. In July 2015, there were 81 different Pseudomonas species that had at least one genome sequence deposited in GenBank, along with another 125 genomes that were designated “Pseudomonas species,” followed by a strain name. Members of the Pseudomonas genus are highly adaptable, as evidenced by their successful colonization of many different environments and their great deal of metabolic versatility and genetic plasticity (1). They have been isolated from various sources, including water, soil, plants, animals, and humans. Many isolates are of interest as possible biocontrol agents, in particular, Populus-associated strains that promote plant growth, increase disease resistance, and improve phytoremediation potential (2).

Populus root systems (e.g., Populus deltoides), host to diverse microbial communities (3, 4), have become an ideal model in which to study plant-microbe interactions both in (endosphere) and on or near (rhizosphere) roots because of the relatively fast growth of trees as a potential woody biomass crop. Moreover, genetic tools are available and the genome of one species (Populus trichocarpa [western balsam poplar]) has been sequenced (5). The 16S rRNA gene pyrosequencing of microbial communities associated with native P. deltoides (eastern cottonwood tree) roots found that Pseudomonas species are enriched in both rhizosphere and root endosphere samples, and this genus was a dominant member of the endophyte community (3). In an ongoing effort to study interactions between bacteria and woody perennials of the fast-growing Populus genus, 21 Pseudomonas strains isolated from endosphere and rhizosphere samples of native P. deltoides roots have been sequenced and characterized as part of the Plant-Microbial Interface (PMI) project in our laboratory (6). The resulting sequences provide insight into the interactions between woody plants and Pseudomonas spp., complementing and expanding the growing number of genomes of pseudomonads that interact with plants. Further, these genomes and comparative studies expand the observed diversity in all Pseudomonas genomes.

Perhaps not surprisingly, diverse phenotypes within the genus Pseudomonas are found to originate from highly diverse genomes, even among closely related isolates (7, 8). This phenotypic and genetic diversity complicates the process of identification to the species level. Indeed, approximately 10% of the sequenced Pseudomonas genomes available at the National Center for Biotechnology Information (NCBI) are not resolved to the species level. A more precise identification of the species present in a given environment would allow a better understanding of their ecological significance.

In this study, a comparative genome analysis based on average amino acid identity (AAI) was performed with >1,000 publicly available Pseudomonas genomes, covering 58 described species and including the 21 Populus-associated PMI genomes. We defined robust and reproducible genomic clusters that correlated with Pseudomonas species in some, but not all, cases. Both clusters containing a mixture of species and species divided over multiple clusters were observed. The global relationships of the genomic clusters on an AAI-based phylogenetic tree provided novel insights into the diversity of Pseudomonas species and showed a phylogenetic homogeneity of Pseudomonas genomic taxonomy-resolving species that was not observed by 16S rRNA gene sequence analysis alone. Observations on the diversity of the species of the genus Pseudomonas were strengthened by pangenome and core genome analyses. Furthermore, the functional contribution of relatively conserved genes of genomic clusters was examined in comparison with the functional distribution of nonconserved genes. Focusing on the Populus-associated PMI strains, pan and core genome analysis was performed to identify genes specific to them, and their relationship was investigated by determining pathway profiles and comparing them with the AAI-based phylogenetic relationship.

## MATERIALS AND METHODS

### Isolation and sequencing of Pseudomonas strains associated with P. deltoides roots.

Strains were isolated from the roots of native P. deltoides trees collected in central Tennessee (36°6′N, 85°50′W) and immediately processed for isolation in October 2009 as described previously (6). Ninety isolates similar to Pseudomonas fluorescens were subjected to multilocus sequence typing. The isolates were all found to belong to the single most dominant operational taxonomic unit in Populus endosphere environmental rRNA samples (3) and originate from the same root endosphere and rhizosphere samples. Each isolate was restreaked, and single-colony stabs were resuspended in 200 μl of water and boiled for 10 min. These cellular lysates were used for PCR amplification of partial *rpoD* and *gyrB* genes as described previously (9). Bidirectional sequencing of the amplicons was performed on an ABI 3730 DNA analyzer. Sequences were trimmed, assembled, and edited to generate a consensus for each isolate in Geneious (v6.1) (10) and aligned with MUSCLE (11), and phylogenetic analysis was conducted with PHYML (12) iterated with 100 bootstrap replications. Twenty-one isolates were chosen to represent the phylogenetic diversity of the overall group of isolates and then selected for whole-genome sequencing (see Fig. S1 in the supplemental material), from which high-quality draft genomes were obtained (6). These genomes ranged in size from 5.8 to 7.3 Mb and contained 155 to 476 scaffolds. None of the isolates showed evidence of plasmids. The isolates contained between 5,384 and 6,334 genes, resulting in an average of 88.4% coding sequence. For a summary of the genomic characteristics of these 21 strains, see reference 6.

### Pseudomonas data set.

On 17 December 2014, we downloaded all of the publicly available Pseudomonas genomes, including the 21 PMI genomes (selected as described above) sequenced as part of the Oak Ridge National Laboratory PMI project (6), from GenBank at the NCBI. The complete data set included 733 genomes with a status of “contig,” 243 with a status of “scaffold,” 16 with a status of “chromosome with gaps,” 16 with a status of “chromosome,” and 65 with a status of “complete genome.” These 1,073 genomes were organized into 58 described species plus 96 Pseudomonas isolates not classified to the species level by NCBI taxonomy (for a summary, see Table S1 in the supplemental material). For Pseudomonas proteomes, we extracted coding sequences and their products from GenBank files where available (440 GenBank files). For genomes without GenBank protein files, we ran Prodigal (13) to annotate and predict protein sequences. P. deltoides root-associated Pseudomonas strains were designated GMXX, where GM25, GM48, GM49, GM74, and GM84 were isolated from the rhizosphere and the rest were from the endosphere. The analysis shown in Fig. S2 in the supplemental material includes 21 additional P. fluorescens genomes released at NCBI between December 2014 and March 2015.

### Average AAI and phylogenetic analyses.

The first analysis comprised pairwise comparisons of the AAIs (14) of the 1,073 Pseudomonas genomes plus one Cellvibrio japonicus genome and one Azotobacter vinelandii genome. For every pair of Pseudomonas genomes, we first identified reciprocal conserved protein-coding genes with UBLAST (R. C. Edgar, unpublished data) with cutoffs of 30% sequence identity and a minimum of 70% alignment length of the query sequence. The AAI was based on reciprocal identities of conserved protein-coding genes and is not symmetrical. The average of the two AAI values was assigned to the pair. Genomic clusters were generated with an AAI cutoff of 95% such that members of different genomic clusters cannot not have an AAI of >95% and members within the genomic clusters have paths consisting of edges connecting Pseudomonas isolates with an AAI of >95%. This cutoff has been previously shown to correspond to the 70% DNA-DNA hybridization (DDH) used for bacterial classification at the species level (14). The AAI tree was built with BIONJ (15) to dissimilarities of AAI values (100% minus AAI). In the tree obtained, branches below the nodes were collapsed when all of the members below the nodes belonged to the same genomic cluster.

### 16S rRNA gene sequence and phylogenetic analyses.

RNAmmer v1.2 (16) was used to obtain the full 16S rRNA gene sequence of each genome. Only 16S rRNA genes that were >1,400 nucleotides (nt) long were considered. In cases where multiple copies of the 16S rRNA gene are present in a genome, the 16S rRNA gene that was closest to 1,500 nt long was selected. This resulted in 116 Pseudomonas genomes without full-length 16S rRNA genes. We aligned 16S rRNA genes by using MAFFT (17) and applied FastTree (18) by using a combined generalized time-reversible model and a single rate for each site (GTR+CAT model) to approximate a 16S rRNA gene-based maximum-likelihood tree.

### Pangenome and core genome analyses of Pseudomonas isolates.

Protein-coding gene families were generated by USEARCH (19) by using members of each family with at least 50% sequence identity for at least 50% of the length of the query sequence. The pangenome of a given data set is defined by all of the combined protein-coding gene families found in that data set, and its size (called the pangenome size) is defined as the total number of protein-coding gene families therein. Since only 6% of the Pseudomonas isolates are completely sequenced and unknown portions of genes are missing from 94% of the genomes, a core genome of a given data set is defined as all of the protein-coding gene families found in 95% of the Pseudomonas isolates considered. The singleton genome consists of protein-coding gene families unique to Pseudomonas isolates. Protein-coding gene families that are neither core nor singleton families were defined as accessory genes.

### Functional analysis of core and accessory genes.

A wrapper script (pfam_scan.pl) provided by the Sanger Institute was used to assign Pfam families to centroids of protein-coding gene families of core and accessory genes for each genomic cluster (clusters of Pseudomonas isolates by AAI with a 95% cutoff). Then Gene Ontology (GO) terms were assigned on the basis of mapping of GO terms to Pfam entries supplied by InterPro for InterPro2GO mapping. The assigned GO terms were converted to their parent GO terms at the second level on a GO graph (go-basic.obo obtained from http://geneontology.org/page/download-ontology). The frequencies of functional terms were normalized by the total occurrence in each functional category (biological process, molecular function, cellular component). Functional terms showing statistically significant differences between core and accessory genes were identified by Mann-Whitney-Wilcoxon test with a *P* value of 0.00001 in R.

### Pathway profile analysis of Populus-associated Pseudomonas isolates.

Pathway profiles were generated for genomes annotated by KEGG Orthology (KO) terms with the KAAS pipeline (20). Pathways were mapped by using their abundance, which was the accumulated frequency of KO terms involved in pathways where only binary information of KO terms based on the KEGG pathway map (found in ko00001.keg) were considered.

## RESULTS AND DISCUSSION

### Genomic clusters of Pseudomonas isolates by AAI.

**FIG 1:**
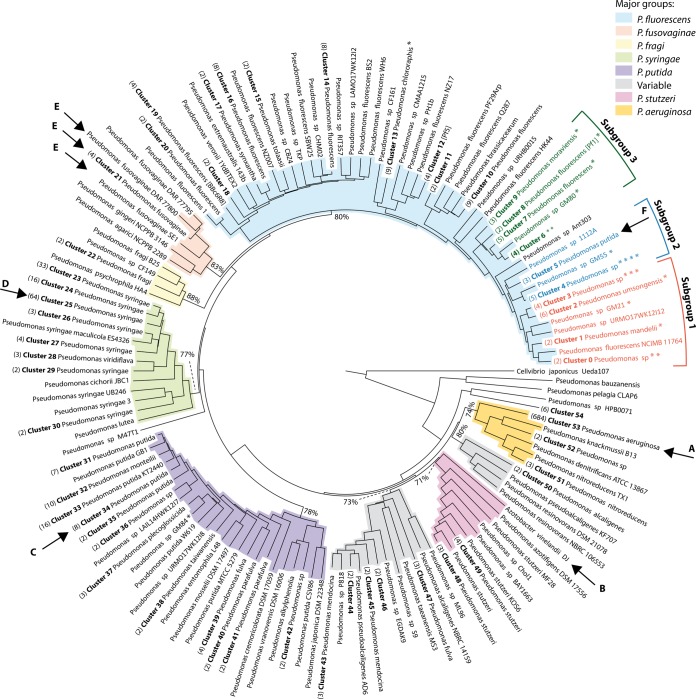
AAI-based tree of 1,073 Pseudomonas genomes with collapsed branches for more than one genome per genomic cluster. The tree was rooted to C. japonicus. Genomic clusters are numbered (in bold), the number of genomes included in each cluster (when it is more than one) is shown in parentheses, and the dominant species name appears after the cluster number. Genomic clusters containing single genomes (singleton clusters) are identified by the strain name. The asterisks represent the proportions of Pseudomonas isolates sequenced as part of the Oak Ridge National Laboratory through the PMI project that are included in particular clusters. Nine major groups corresponding to major species groups in the genus Pseudomonas are color coded, and the minimum AAI values (percentages) of these major groups are shown at the last common ancestor nodes. Arrows A to F indicate clusters that are discussed in the text.

Figure 1 shows an AAI tree of 1,073 Pseudomonas genomes rooted to C. japonicus. The genomes fall into nine major groups corresponding to major Pseudomonas species groups, which are color coded in Fig. 1, including the five well-known major groups of Pseudomonas aeruginosa, P. fluorescens, Pseudomonas putida, Pseudomonas stutzeri, and Pseudomonas syringae (21); two groups comprising the Pseudomonas fragi and Pseudomonas fusovaginae genomes, respectively; and two groups containing a mixture of known and unknown species. The minimum AAI values of the major groups are indicated at the last common ancestor nodes. Each of the nine major groups contains distinct genomic clusters, many of which correspond to species. The 1,073 Pseudomonas genomes were assigned to a total of 131 genomic clusters, of which 54 contain more than 1 genome and the rest contain only a single genome (for a summary, see Table S1 in the supplemental material). In Fig. 1, genomic clusters with two or more genomes were collapsed into a single branch for legibility. For an uncollapsed version of the tree in Fig. 1, see Fig. S3 in the supplemental material. Species names (as recorded by the NCBI) with the most abundance within the genomic clusters were added to genomic cluster labels. Thus, the label “cluster 53 P. aeruginosa” (arrow A in Fig. 1) indicates that all of the members of that branch came from cluster 53 and P. aeruginosa is the most abundant species in cluster 53. Strain names were used to label branches corresponding to singleton clusters. The asterisks represent branches containing Populus-associated PMI Pseudomonas isolates. At this resolution, the Populus-associated isolates contribute to 14 genomic clusters, highlighting the diversity of Pseudomonas isolates associated with Populus treess. Table S1 in the supplemental material lists the 1,073 Pseudomonas genomes from this study with the assembly ID, organism name, GC content, sequencing status, sequencing center, and genomic cluster shown in Fig. 1.

By far the most common species represented in the 1,073 genomes was P. aeruginosa (a species that belongs to the most frequently encountered opportunistic human pathogens). It is represented by 680 of the genomic sequences in cluster 53. Eight other species were represented by more than five genome sequences: P. syringae (comprising mainly plant pathogens) with 119 genomes, P. putida (studied for biodegradation and biosynthesis capabilities) with 32 genomes, P. fluorescens (plant growth-promoting rhizobacteria) with 29 genomes, P. chlororaphis (a species with growth-promoting and antipathogen activities for a number of plant species) and P. stutzeri (a soil inhabitant with bioremediation activity) with 9 genomes each, Pseudomonas mendocina (alginate production and biodegradation) with 7 genomes, and Pseudomonas fuscovaginae (an economically important rice pathogen) and Pseudomonas monteilii (a species of interest for biodegradation and biosynthesis) with 6 genomes each. Twenty-nine species were represented by singleton genomes, while 96 Pseudomonas genomes had no species name listed in the NCBI taxonomy. Additional Pseudomonas species have been defined that did not have a sequenced genome at the time of this analysis and were not included in this study.

Notable observations based on genomic clusters and the AAI tree of 1,073 Pseudomonas genomes are summarized in the following.

(i) We compared our genomic clusters to other grouping efforts by NCBI and the Integrated Microbial Genomes (IMG) system. At the NCBI, genomes are grouped in cladeIDs on the basis of conserved universal protein sequences (according to personal communication with NCBI staff). IMG groups genomes on the basis of cliques generated by average nucleotide sequence identity (ANI) and the alignment fraction (22). Compared to the 131 clusters in this study, there are about half as many cladeIDs and about twice as many cliques of Pseudomonas genomes. All of the members of our genomic clusters with at least two members belonged to groups with the same NCBI cladeIDs. At any given time point, the Pseudomonas data set in the IMG database is never completely identical to the Pseudomonas data set downloaded from the NCBI, making comparisons only approximate. However, no discrepancies were observed between clusters in this study and cliques defined by IMG in terms of Pseudomonas species composition. Therefore, issues concerning the naming of species, as discussed below, are supported by two additional independent methods.

(ii) The species P. aeruginosa is part of a single tight genomic cluster composed of 684 genomes. These are listed in Table S1 in the supplemental material and are also available as a zoomable tree (http://dtree.ornl.gov/pseudo_final_aai.html). The monophyletic group on the AAI tree in Fig. 1 (cluster 53, arrow A in Fig. 1) contains not only 680 P. aeruginosa genomes but also one Pseudomonas otitidis genome and three Pseudomonas sp. genomes. On the basis of pairwise AAI comparisons, the tight local grouping of the cluster 53 genomes combined with their global relationship on the AAI-based tree suggests that all of these genomes belong to the species P. aeruginosa (with AAI of >95%). The only other species found in this cluster is P. otitidis, which is represented by a single genome sequence available thus far. It showed high (98.6%) 16S rRNA gene sequence identity but a low (40%) DDH with P. aeruginosa, which was the basis for proposing P. otitidis as a novel species (23). However, this P. otitidis genome has 99.4% ANI (24) to several of its neighbors on the AAI tree (P. aeruginosa strains BWHPSA020, BWHPSA023, and PA21_ST175). In combination, these AAI and ANI results indicate the need to revisit the naming of P. otitidis, as its genomic signature closely resembles that of P. aeruginosa.

(iii) P. fluorescens genomes are quite diverse. In contrast to the >600 P. aeruginosa genomes that all fit in cluster 53 in Fig. 1, the 29 genomes recorded under the name P. fluorescens at the NCBI display a high degree of phylogenetic heterogeneity, as they are found in 20 different genomic clusters scattered throughout the large P. fluorescens group (light blue in Fig. 1), with intercluster AAIs of <95%. Although a few sequenced P. fluorescens isolates have been well characterized, for most sequenced isolates of this species, little or no phenotypic data are available, so that a correlation between phenotype and genomic clustering could not be assessed. There was no association between clustering and the host species or source from which the isolates were derived or their geographic origin (data not shown).

(iv) The Azotobacter genome is found in the P. stutzeri group. The A. vinelandii DJ genome was included in this analysis because previous findings, based on comparative genomics of Azotobacter and Pseudomonas (25), showed that it was closely related. Its position is indicated by arrow B in Fig. 1 and is consistent with inclusion in the P. stutzeri group. Many species named on the basis of their 16S rRNA gene sequences do not yet have a sequenced genome and therefore have not been subjected to genome analysis. As with Azotobacter, it is possible that additional genera may be more closely related to the species now part of the Pseudomonas genus and some Pseudomonas species are not as closely related as once thought.

(v) Cluster 34 showed the greatest species complexity (arrow C in Fig. 1), as it includes members of four difference species, Pseudomonas monteilii, Pseudomonas plecoglossicida, P. putida, and Pseudomonas taiwanensis. The name P. putida was assigned to the collapsed branch because P. putida was the most abundant species. Clearly, these data support a reevaluation of these species, as has been suggested before, on the basis of phenotypic characteristics (26).

(vi) P. avellanae and P. syringae genomes are in a single, tight cluster. All three of the P. avellanae genomes included in the analysis grouped together in the middle of cluster 25 (arrow D in Fig. 1), which contained primarily P. syringae genomes. Independent analyses reviewed elsewhere also place P. avellanae in the middle of a clade corresponding to P. syringae, suggesting that redefinition of the species P. avellanae is needed (27).

(vii) The six P. fuscovaginae genomes are in three different clusters, which are positioned next to each other and form a monophyletic group on the AAI tree (arrows E in Fig. 1). The local grouping based on pairwise AAI comparison combined with their global relationship on this whole-proteome-based tree still suggests that P. fuscovaginae consists of three closely related clades, perhaps subspecies, that are not resolved by 16S rRNA gene sequence analysis (see Fig. S4 in the supplemental material). The same circumstantial evidence was observed with Pseudomonas nitroreducens (positioned in the opposite part of the tree, a major group that also contains P. aeruginosa).

(viii) Thirty-two P. putida members are organized into 11 genomic clusters. One of these, cluster 5 (arrow F in Fig. 1), is positioned within the P. fluorescens major group. On the basis of a variety of genomic information, including 16S rRNA gene sequences, one of the “P. putida” genomes (GCA_000729805) of cluster 5 in the P. fluorescens group is not classified properly.

### Pangenome and core genome analyses of all Pseudomonas isolates.

**FIG 2:**
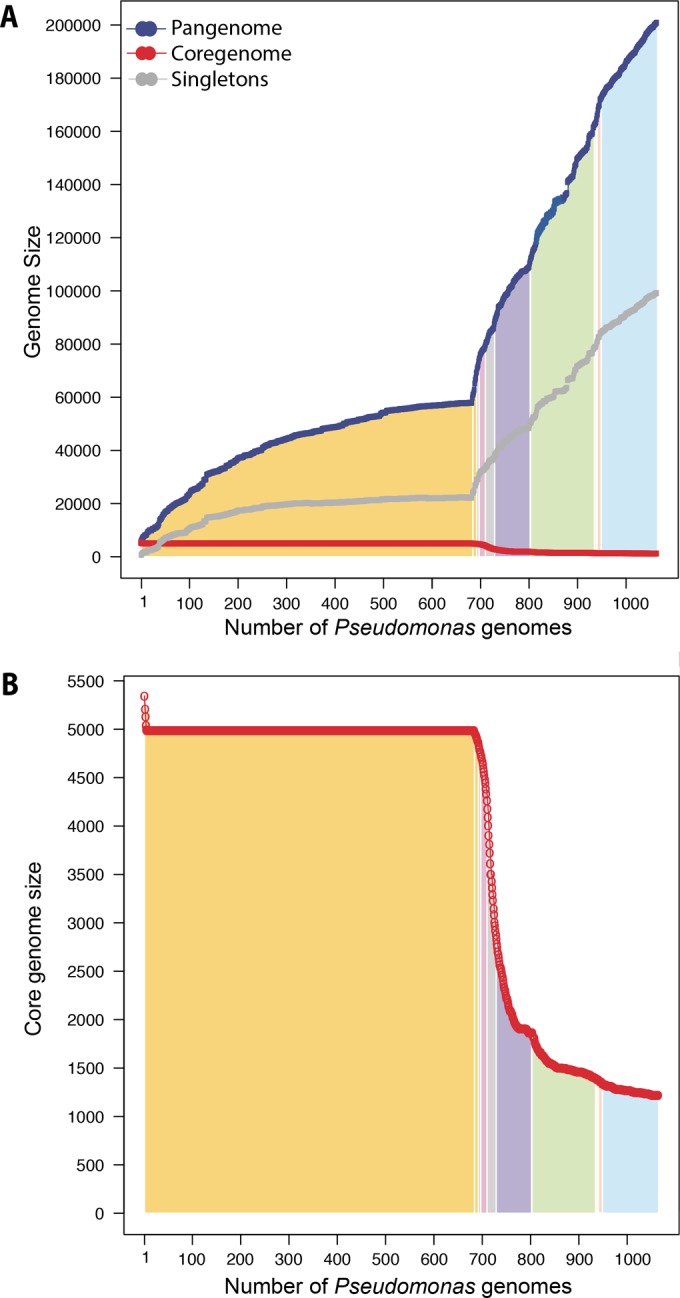
Pangenome, core genome, and singleton genome analyses. (A) Accumulative pangenomes (blue), core genomes (red), and singleton genomes (gray) when Pseudomonas isolates were added in the order of their positions on the AAI tree in Fig. 1, starting with genomes from the node labeled “cluster 53 P. aeruginosa.” The color-coded areas correspond to the major groups defined in Fig. 1. (B) The core genome of panel A plotted on a zoomed scale.

We performed pangenome and core genome analyses by adding Pseudomonas genomes in the order of their phylogenetic locations (derived from Fig. 1), starting with genomes from the node labeled “cluster 53 P. aeruginosa,” the largest species group, and going clockwise. In Fig. 2A, the *y* axis represents the sizes of pangenomes, core genomes, and singleton genomes. The area below the plot is shaded according to the major groups defined in Fig. 1. The genomes included here display a large amount of genetic diversity, with an increasing pangenome size totaling 200,839 protein-coding gene families and 99,176 singleton protein-coding gene families. The pangenome curve has not saturated, indicating that there is significant diversity still undiscovered in the Pseudomonas genus. This study, along with several past studies of Pseudomonas (7, 8, 28), indicates the need to sequence multiple isolates, especially in the P. fluorescens group, in order to study functional capabilities from a pangenomic perspective. The total core genome contains 1,224 protein-coding gene families (Fig. 2B). Interestingly, even though the pangenome suggests that there is much more diversity, the core genome appears to be plateauing at a little bit more than 1,000 protein-coding gene families. The pangenome and core genome analyses set P. aeruginosa apart from other Pseudomonas species in genomic relatedness. The core genome of P. aeruginosa (cluster 53) quickly reaches a plateau and is not influenced by the addition of the P. otitidis genome. This might be further support for a reexamination of the species description of this isolate.

### Functional analysis of core and accessory genes for genomic clusters.

**FIG 3:**
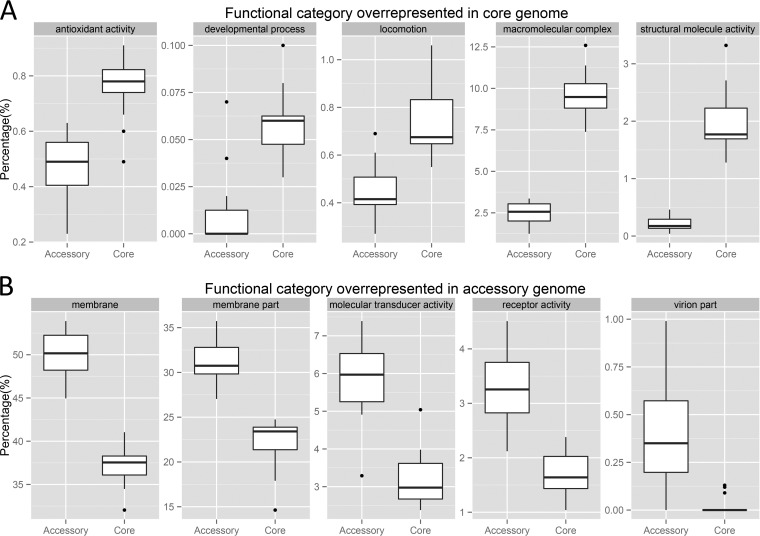
Box-and-whisker plots of functional terms overrepresented in core genes compared to accessory genes (A) and accessory genes compared to core genes (B) among genomic clusters with at least five members. The box plots shown were drawn with the upper and lower lines corresponding to the first and third quartiles, the middle lines corresponding to the medians, and the upper and lower whiskers extended to 1.5 times the interquartile ranges. Black dots are outliers outside 1.5 times the interquartile range. Note the difference in scale between the panels.

To compare the functional roles of relatively conserved genes and variable genes in the genus Pseudomonas, genomic clusters with at least five members (for a summary, see Table S2 in the supplemental material) were analyzed by comparing the functional profiles of core genes with those of accessory genes. First, GO terms at the second level of each functional category (biological process, molecular function, and cellular component) were assigned to core and accessory genes for each genomic cluster (mapping of GO terms to core and accessory genes is described in Materials and Methods). The abundance of functional terms was normalized by the total number of functional terms considering functional categories separately. Figure 3A represents those functional categories that showed a significant abundance of core genes compared to the accessory genes in the genomic clusters. These genes were related to antioxidant activity, developmental processes, locomotion, macromolecular complexes (whose definition by GO is a stable assembly of two or more macromolecules, for example, proteins, nucleic acids, carbohydrates, or lipids, in which the constituent parts function together), and structural molecule activity (defined by GO as the action of a molecule that contributes to the structural integrity of a complex or assembly within or outside the cell). With the exception of genes related to antioxidant activity, all of these functions require intricate interplay of multiple factors, which may be the reason why they are more conserved and thus are found as part of the core genome. Figure 3B summarizes the results of a comparison of overrepresented accessory genes and core genes. Two of these five GO terms relate to membrane components, consistent with variable survival strategies of the cells. For example, bacteria need to avoid viral attacks, for which the surface proteins need to be varied and distributed in the accessory genes. Two important functional categories related to signaling, and mediated at least in part by surface proteins, are also more abundant in the accessory genome. Phage-derived proteins (virion part in Fig. 3B) were almost never found as the core because these genes are typically part of the variable gene content, along with plasmids.

### Populus-associated Pseudomonas isolates. (i) Species classification by AAI.

The membership of PMI Pseudomonas isolates not classified at the species level was summarized by AAI (see Table S3 in the supplemental material). Of the 21 PMI Pseudomonas isolates, 13 were novel genomic clusters in the sense that they did not cluster with members of the recognized Pseudomonas species and did not receive a species assignment, indicating the diversity of sequenced Populus-associated Pseudomonas isolates. GM17 grouped with P. chlororaphis, GM25 and GM30 grouped with P. fluorescens, GM41 grouped with P. mandelii, and GM78 grouped with P. putida. The rest were assigned to genomic clusters composed of more than one Pseudomonas species.

### (ii) The P. fluorescens group.

**FIG 4:**
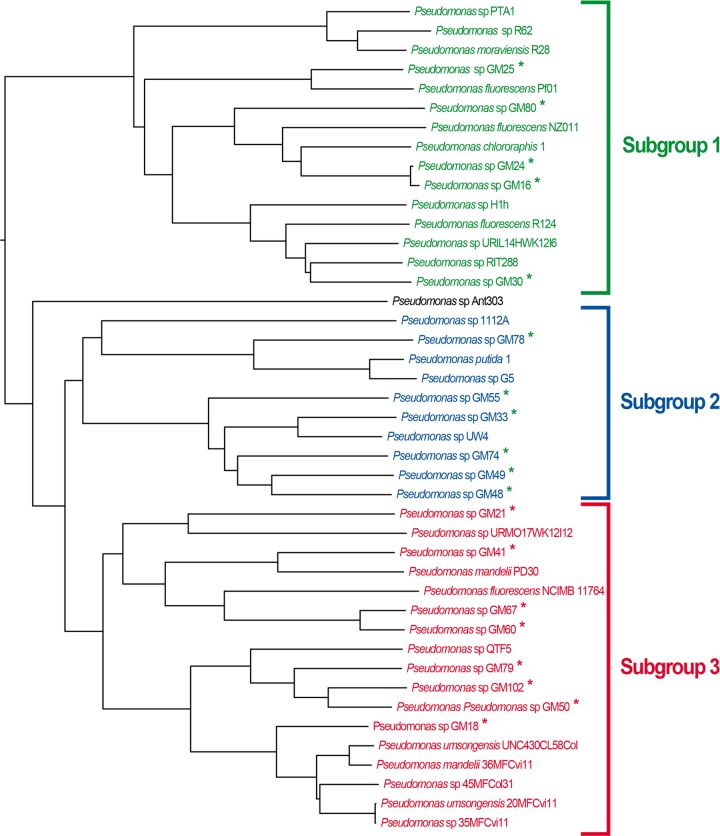
AAI-based tree of 19 Populus-associated Pseudomonas isolates and related Pseudomonas strains. The tree shown was parsed from the tree in Fig. 1 and remained uncollapsed. The 19 Populus-associated isolates, identified by asterisks, form three distinct subgroups.

The largest group in terms of breadth of phylogenetic distance, making up nearly a third of the Pseudomonas tree, is the P. fluorescens group, shown at the top of Fig. 1. As indicated by the asterisks in Fig. 1, 19 of the 21 Populus microbiome Pseudomonas isolates (labeled Pseudomonas sp. GMXX) fall into three distinct subgroups within the P. fluorescens major group, with the other two clustering with P. chlororaphis and P. putida, respectively. An uncollapsed version of this section of the tree is shown in Fig. 4. Forty-three genomes are clustered in the three subgroups including the P. deltoides isolates. These three subgroups extend the known diversity within the group that was originally defined as “subgroup 2” by Loper et al. in 2012 (8). Strain GM25 isolated from the P. deltoides rhizosphere is grouped with P. fluorescens PfO-1, a previously sequenced soil isolate that falls within subgroup 1, and the other three rhizosphere isolates (GM48, GM49, GM74) form a monophyletic group within “subgroup 2.” As described in Materials and Methods, 21 additional P. fluorescens genomes were submitted to GenBank after completion of the phylogenetic analysis. We observed the same three subgroups conserved with these additional genomes (see Fig. S2 in the supplemental material, where seven newly sequenced P. fluorescens isolates fall into these three subgroups). These analyses reveal the genomic diversity of Pseudomonas isolates that can be found in association with Populus species. Further, these results suggest that the isolates may be more appropriately defined as multiple species or a species complex, as suggested in previous phylogenomic studies (7, 8).

### Pangenome and core genome analyses of three Populus-associated subgroups.

Traits that are found in all of the members of distinct subgroups but differentiated from traits that can be expected in only a small subset of isolates would be useful when choosing Pseudomonas strains for use to promote growth and increase disease resistance in plants. To determine what genomic content accounts for the differences between the subgroups, pangenome and core genome analyses of the three subgroups shown in Fig. 4 were performed in order to identify genes that are present in all of the members of a particular subgroup and absent from the remaining subgroups. A numerical overview is summarized in Table 1. The three distinct subgroups have similar-size core genomes. The distributions of the GO terms of their core genes, with second-level terms, are also very similar (data not shown). When combining subgroups 1 and 2, the core genome contains 109 more core genes than the combination of subgroups 1 and 3 and 56 more than subgroups 2 and 3 combined. However, subgroups 2 and 3 combined have 22 core genes specific to these subgroups, whereas other pairs of subgroups do not have a single core gene specific to them, which may cause subgroups 2 and 3 to merge first and then subgroup 1 in Fig. 4. Subgroup 1 has 20 genes (among 3,775 core genes) specific to it (and thus not present in subgroups 2 and 3). These include several sensory systems acting in two-component signal transduction, a TonB-dependent receptor, and a phosphorelay sensor. Other proteins encoded by genes found only in subgroup 1 include a PAP2 superfamily, a glucose 1-dehydrogenase, an FeS assembly protein, a radical SAM family protein, and a heme oxygenase. Subgroup 2 contains only two specific genes (among 3,546 core genes) annotated as “hypothetical.” Subgroup 3 has a single gene specific for this subgroup, annotated as a “hydrolase enzyme” (GO:0016787). For a summary of the subgroup-specific genes with their available GO annotations, see Table S4 in the supplemental material.

**TABLE 1:** Pangenome, core genome, and singleton genome analyses of Populus-associated subgroups

Analysis	No. of genes in:					
	Subgroup 1	Subgroup 2	Subgroup 3	Subgroups 1 and 2 combined	Subgroups 1 and 3 combined	Subgroups 2 and 3 combined
Pangenome	12,871	13,011	15,216	19,908	21,120	20,480
Core genome	3,775	3,546	3,269	3,096	2,987	3,040
Unique genome	20	2	1	0	0	22

### Pathway profile analysis.

The Populus-associated Pseudomonas isolates and their phylogenetically related isolates from the three subgroups (Fig. 4) were further compared by using pathway profiles. Note that GM17 and GM84 are not in the three subgroups, so they were not included in this analysis. The observed pathway frequencies (see Fig. S5 in the supplemental material) are quite similar across the three subgroups. The subgroups defined by hierarchical clustering based on pathway profiles remained largely intact, supporting whole-genome-based relationships of Populus-associated Pseudomonas isolates. The two exceptions are (i) that GM21 is in subgroup 3 on the basis of genomic relatedness by AAI but is grouped with members of subgroup 2 on the basis of pathway profiles and (ii) that GM80, a subgroup 2 member on the basis of AAI, is grouped with members of subgroup 3 on the basis of pathway profiles. Nevertheless, by and large, the strong conservation of functional protein pathways supports the genomic clustering based on AAI.

## Supplementary Material

Supplemental material
