# Supplementary material for: Diversity of Pseudomonas Genomes, Including Populus-Associated Isolates, as Revealed by Comparative Genome Analysis
Source: Appl Environ Microbiol. 2015 Dec 22;82(1):375–83. doi: 10.1128/AEM.02612-15 (PMC4702629; doi:10.1128/AEM.02612-15)
Supplement: Supplemental material [file supp_82_1_375__index.html]

Diversity of Pseudomonas Genomes, Including Populus-Associated Isolates, as Revealed by Comparative Genome Analysis — Supplemental material 

# Diversity of Pseudomonas Genomes, Including Populus-Associated Isolates, as Revealed by Comparative Genome Analysis

## Supplemental material

- Supplemental file 1 -

  Phylogenetic analysis of partial *rpoD* genes found in *Pseudomonas* isolates (Fig. S1), AAI-based trees (Fig. S2 and S3), 16S rRNA-based tree (Fig. S4), pathway profile analysis (Fig. S5), genomic clusters with at least five members (Table S2), and membership of PMI *Pseudomonas* isolates into genomic clusters (Table S3).

  PDF, 1.3M
- Supplemental file 2 -

  *Pseudomonas* data set (Table S1).

  PDF, 233K
- Supplemental file 3 -

  Subgroup-­specific genes with annotation (Table S4).

  XLSX, 65K
