# Supplementary material for: Diversity of Pseudomonas Genomes, Including Populus-Associated Isolates, as Revealed by Comparative Genome Analysis
Source: Appl Environ Microbiol. 2015 Dec 22;82(1):375–83. doi: 10.1128/AEM.02612-15 (PMC4702629; doi:10.1128/AEM.02612-15)
Supplement: Supplemental material [file AEM.02612-15_zam999116834so1.pdf]

Comparative genome analysis of *Pseudomonas* genomes including *Populus*-associated isolates

Se-Ran Jun[1], Trudy M. Wassenaar[3], Intawat Nookaew[2], Loren Hauser[2], Visanu Wanchai[2], Miriam Land[2], Collin Timm[2], Tse-Yuan S. Lu[2], Christopher W. Schadt[2], Mitchel J. Doktycz[2], Dale A. Pelletier[2], David W. Ussery[2,#]

Running Head: Comparative *Pseudomonas* genome analysis

[1] Joint Institute for Computational Sciences, University of Tennessee, Knoxville, TN, USA

[2] Biosciences Division, Oak Ridge National Laboratories, Oak Ridge, TN, USA

[3] Molecular Microbiology and Genomics Consultants, Zotzenheim, Germany

#Address correspondence to David W. Ussery

Oak Ridge National Laboratory

Biosciences Division

Oak Ridge, TN 37831

Phone: (865) 574-8201

Fax: (865) 574-3555

Email: usserydw@ornl.gov

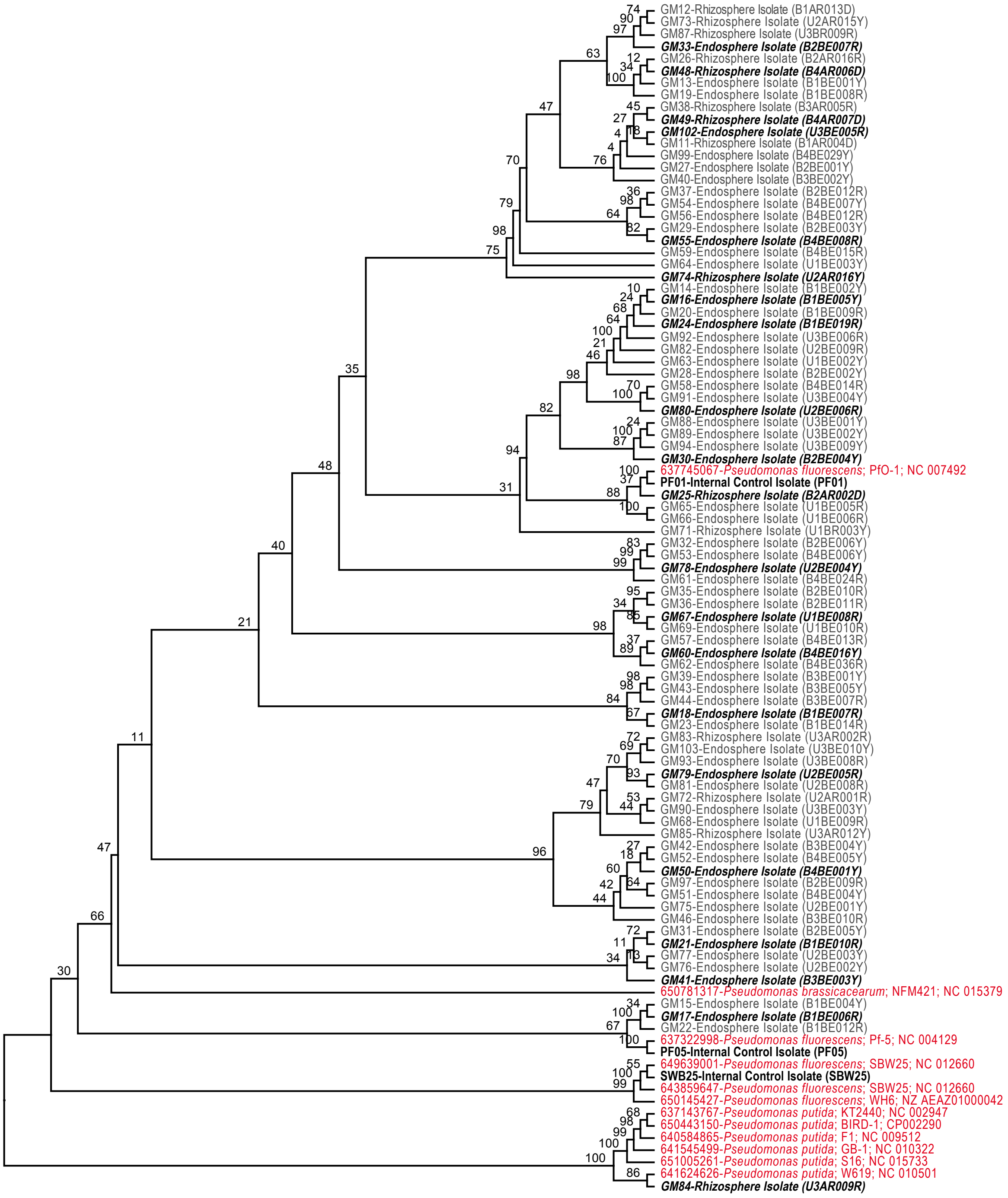

Figure S1. Phylogenetic analysis of partial *rpoD* genes found in isolates of *Pseudomonas* from

Populus deltoides roots and selected references taxa (Maximum likelihood, bootstraps = 100). Genes from reference isolates are listed by their GI number. Strains from the current study are labeled to denote the compartment (endosphere or rhizosphere) and tree from which the isolate originated. Isolates representing diverse and well-supported groups were selected for complete genome sequence analysis, indicated in bold. Reference complete genomes are shown in red.

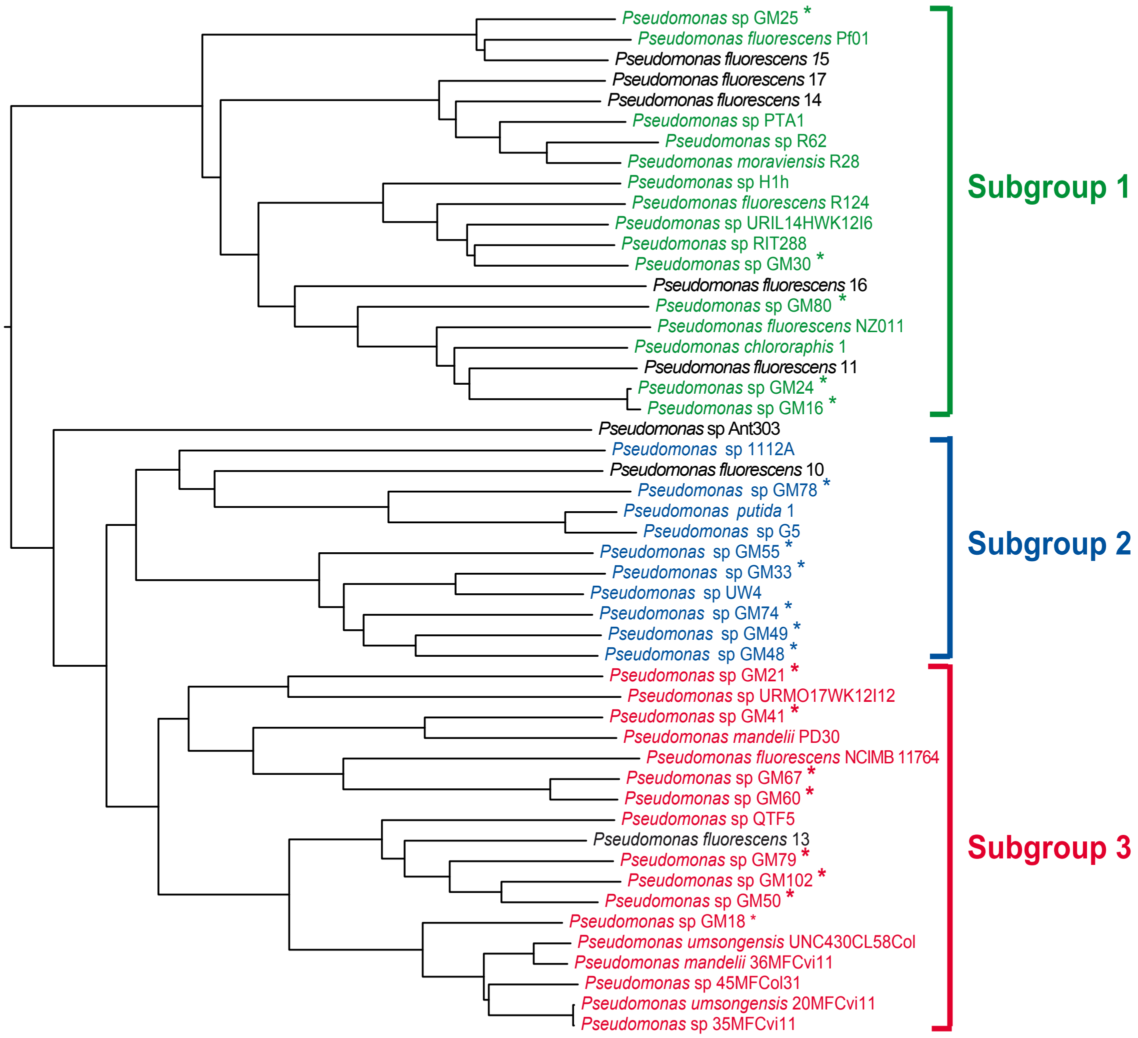

Figure S2. AAI-based tree of 19 *Populus-associated Pseudomonas* isolates and related *Pseudomonas* species with newly sequenced *P. fluorescens* in black.

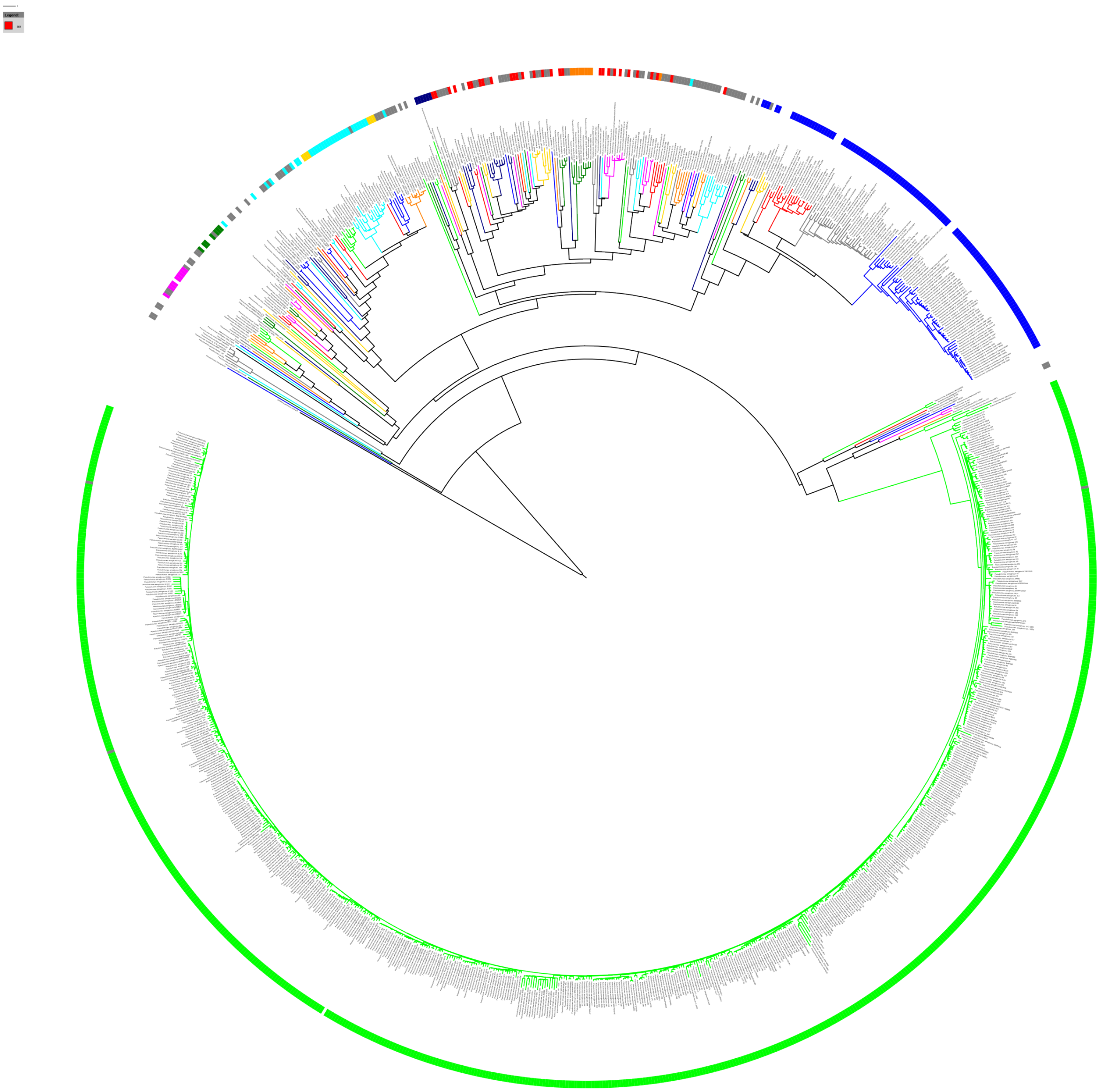

Figure S3. AAI-based tree of 1073 *Pseudomonas* genomes. The outer ring and branches are colored by *Pseudomonas* species groups defined by NCBI taxonomy.

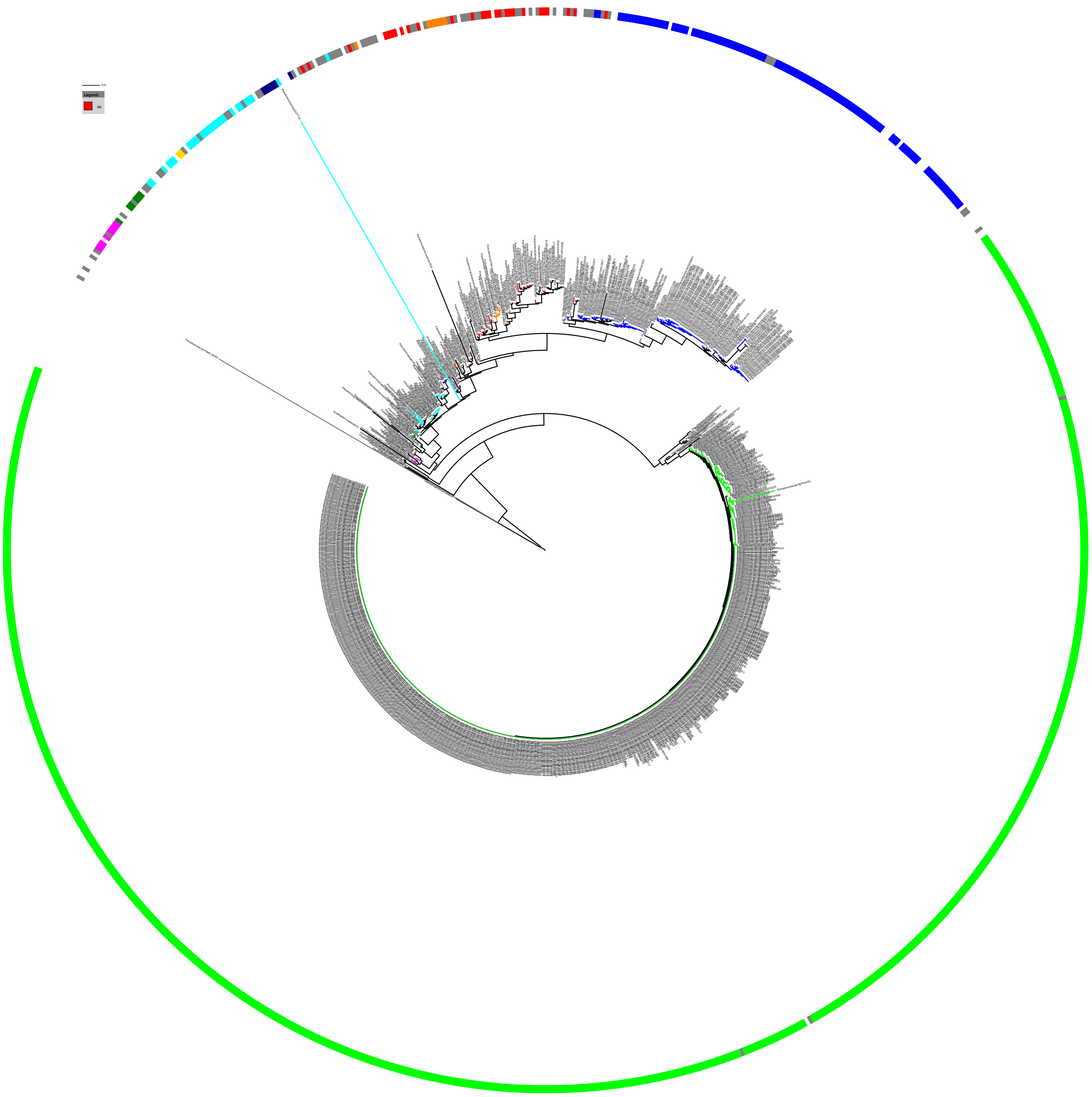

Figure S4. 16S-rRNA based tree. We considered only 16S rRNA genes (extracted by RNAmmer v1.2 (1)) whose length is larger than 1400 nt-long, and chose one 16S rRNA gene whose length is closest to 1500 when the multiple copies of 16S rRNA genes are present in the genomes, resulting in 116 *Pseudomonas* without full-length 16S rRNA genes out of 1073 *Pseudomonas* isolates. We aligned 16S rRNA genes using MAFFT (2), and applied FastTree (3) using a combined model of generalized time-reversible and a single rate for each site (GTR+CAT model)

to approximate a 16S rRNA-based maximum likelihood tree. The outer ring and branches are colored by *Pseudomonas* species groups defined by NCBI taxonomy.

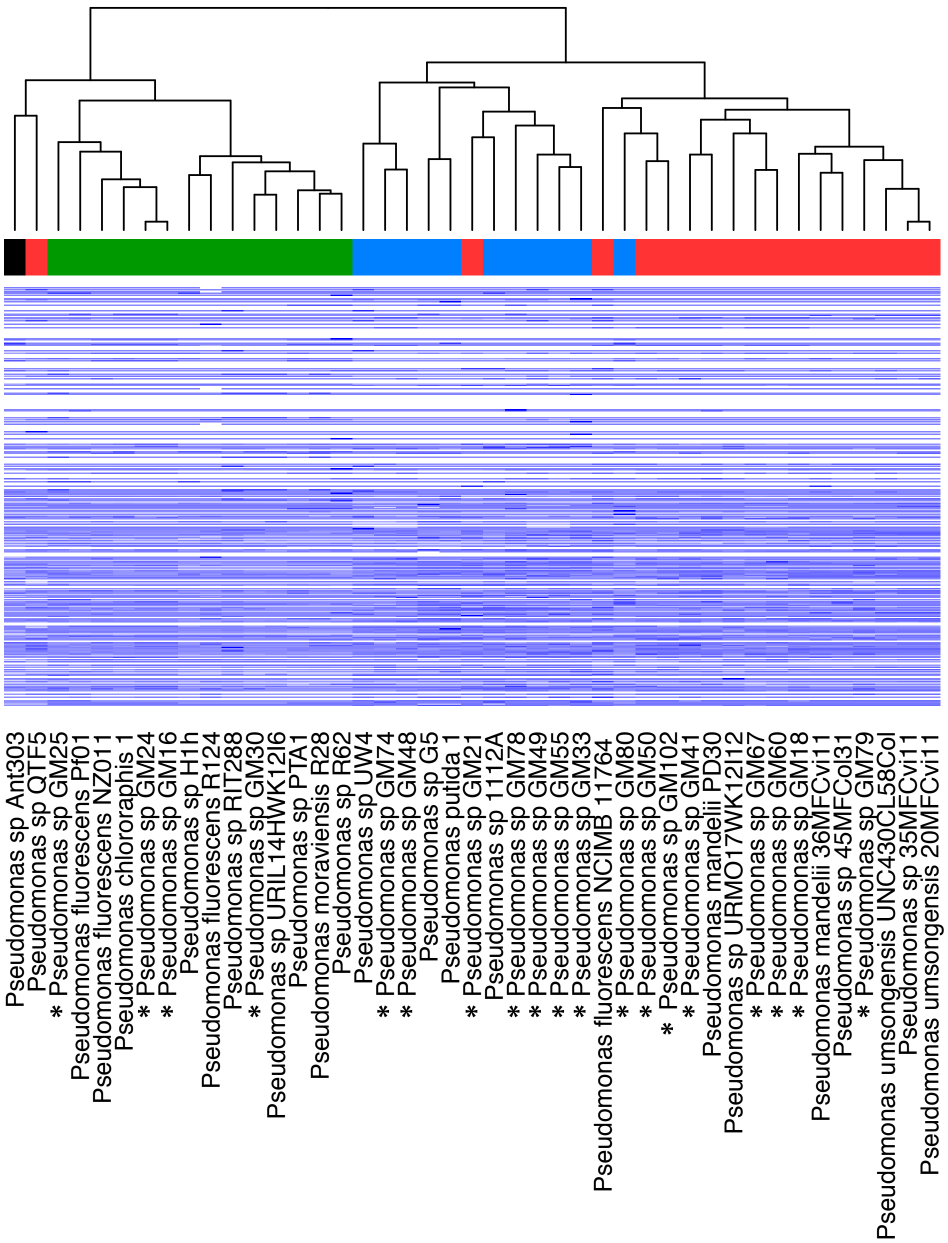

Figure S5. Pathway profiles analysis of PMI *Pseudomonas* isolates. The asterisk indicates PMI *Pseudomonas* isolates. The color code at the X-axis corresponds to three distinct subgroups identified in Figure 2.

Table S1. *Pseudomonas* dataset.

Table S2. Genomic clusters with at least five members.

Genomic cluster Index	Cluster size	Species by NCBI Taxonomy
Cluster_53	684	*Pseudomonas aeruginosa* (680)<br>*Pseudomonas otitidis* (1)<br>*Pseudomonas sp* (3)
Cluster_25	64	*Pseudomonas syringae* (61)<br>*Pseudomonas avellanae* (3)
Cluster_23	33	*Pseudomonas syringae* (33)
Cluster_33	16	*Pseudomonas putida* (15)<br>*Pseudomonas monteilii* (1)
Cluster_24	16	*Pseudomonas syringae* (13)<br>*Pseudomonas savastanoi* (3)
Cluster_32	10	*Pseudomonas monteilii* (3)<br>*Pseudomonas putida* (1)<br>*Pseudomonas sp* (6)
Cluster_10	9	*Pseudomonas fluorescens* (4)<br>*Pseudomonas brassicacearum* (3)<br>*Pseudomonas sp* (2)
Cluster_13	9	*Pseudomonas chlororaphis* (8)<br>*Pseudomonas sp* (1)
Cluster_16	8	*Pseudomonas fluorescens* (4)<br>*Pseudomonas sp* (4)
Cluster_14	8	*Pseudomonas fluorescens* (3)<br>*Pseudomonas simiae* (2)<br>*Pseudomonas sp* (3)
Cluster_34	8	*Pseudomonas putida* (3)<br>*Pseudomonas monteilii* (2)<br>*Pseudomonas plecoglossicida* (1)<br>*Pseudomonas taiwanensis* (1)<br>*Pseudomonas sp* (1)
Cluster_31	7	*Pseudomonas putida* (6)<br>*Pseudomonas sp* (1)
Cluster_54	6	*Pseudomonas psychrotolerans* (1)<br>*Pseudomonas oryzihabitans* (1)<br>*Pseudomonas oleovorans* (1)<br>*Pseudomonas sp* (3)
Cluster_2	6	*Pseudomonas umsongensis* (2)<br>*Pseudomonas mandelii* (1)

		Pseudomonas sp (3)
Cluster_7	5	*Pseudomonas fluorescens* (1)<br>*Pseudomonas sp* (4)
Cluster_4	5	*Pseudomonas sp* (5)

Table S3. Membership of PMI Pseudomonas isolates into genomic clusters.

PMI *Pseudomonas*	Genomic cluster index	Cluster size	*Pseudomonas* species<br>by NCBI taxonomy
GM60	Cluster_0	2	*Pseudomonas sp (*2)
GM67	Cluster_0	2	*Pseudomonas sp* (2)
GM41	Cluster_1	2	*Pseudomonas mandelii* (1)<br>*Pseudomonas sp* (1)
GM18	Cluster_2	6	*Pseudomonas umsongensis* (2)<br>*Pseudomonas mandelii* (1)<br>*Pseudomonas sp* (3)
GM50	Cluster_3	4	*Pseudomonas sp* (4)
GM79	Cluster_3	4	*Pseudomonas sp* (4)
GM102	Cluster_3	4	*Pseudomonas sp* (4)
GM33	Cluster_4	3	*Pseudomonas sp* (3)
GM48	Cluster_4	5	*Pseudomonas sp* (5)
GM49	Cluster_4	5	*Pseudomonas sp* (5)
GM74	Cluster_4	5	*Pseudomonas sp* (5)
GM78	Cluster_5	3	*Pseudomonas putida* (1)<br>*Pseudomonas sp* (2)
GM16	Cluster_6	4	*Pseudomonas chlororaphis* (1)<br>*Pseudomonas fluorescens* (1)<br>*Pseudomonas sp* (2)
GM24	Cluster_6	4	*Pseudomonas chlororaphis* (1)<br>*Pseudomonas fluorescens* (1)<br>*Pseudomonas sp* (2)
GM30	Cluster_7	5	*Pseudomonas sp* (4)<br>*Pseudomonas fluorescens* (1)
GM25	Cluster_8	2	*Pseudomonas fluorescens* (1)<br>*Pseudomonas sp* (1)
GM17	Cluster_13	9	*Pseudomonas chlororaphis* (8)<br>*Pseudomonas sp* (1)
GM55	Singleton	1	*Pseudomonas sp* (1)
GM84	Singleton	1	*Pseudomonas sp* (1)
GM80	Singleton	1	*Pseudomonas sp* (1)
GM21	Singleton	1	*Pseudomonas sp* (1)

Table S4. Subgroup-specific genes with annotation.

REFERENCES

1. **Lagesen K, Hallin P, Rodland EA, Staerfeldt H-H, Rognes T, Ussery DW.** 2007. RNAmmer: consistent and rapid annotation of ribosomal RNA genes. Nucleic acids research **35:**3100-3108.
2. **Katoh K, Standley DM.** 2013. MAFFT multiple sequence alignment software version 7: improvements in performance and usability. Molecular biology and evolution **30:**772-780.
3. **Price MN, Dehal PS, Arkin AP.** 2010. FastTree 2--approximately maximum-likelihood trees for large alignments. PloS one **5:**e9490.
