# Supplementary material for: Diversity of Pseudomonas Genomes, Including Populus-Associated Isolates, as Revealed by Comparative Genome Analysis
Source: Appl Environ Microbiol. 2015 Dec 22;82(1):375–83. doi: 10.1128/AEM.02612-15 (PMC4702629; doi:10.1128/AEM.02612-15)
Supplement: Supplemental material [file AEM.02612-15_zam999116834so2.pdf]

Assembly ID	Genomic cluster	Oragnism name	GC%	Status	Center
GCA_000282435	Cluster_0	Pseudomonas sp. GM67	59.6	Contig	Oak Ridge National Lab
GCA_000282415	Cluster_0	Pseudomonas sp. GM60	59.6	Contig	Oak Ridge National Lab
GCA_000690555	Cluster_1	Pseudomonas mandelii PD30	59.0	Contig	University of Guelph SES
GCA_000282315	Cluster_1	Pseudomonas sp. GM41(2012)	59.0	Contig	Oak Ridge National Lab
GCA_000620285	Cluster_2	Pseudomonas umsongensis UNC430CL58Col	59.3	Scaffold	DOE Joint Genome Institute
GCA_000382025	Cluster_2	Pseudomonas sp. 45MFCol3.1	59.4	Scaffold	DOE Joint Genome Institute
GCA_000381285	Cluster_2	Pseudomonas mandelii 36MFCvi1.1	59.2	Scaffold	DOE Joint Genome Institute
GCA_000377725	Cluster_2	Pseudomonas umsongensis 20MFCvi1.1	59.4	Scaffold	DOE Joint Genome Institute
GCA_000378525	Cluster_2	Pseudomonas sp. 35MFCvi1.1	59.4	Scaffold	DOE Joint Genome Institute
GCA_000282195	Cluster_2	Pseudomonas sp. GM18	59.5	Contig	Oak Ridge National Lab
GCA_000282375	Cluster_3	Pseudomonas sp. GM50	59.0	Contig	Oak Ridge National Lab
GCA_000512695	Cluster_3	Pseudomonas sp. QTF5	58.7	Contig	Tianjin Institute of Industrial Biotechnology, Chinese Academy of Sciences
GCA_000282495	Cluster_3	Pseudomonas sp. GM79	58.8	Contig	Oak Ridge National Lab
GCA_000282555	Cluster_3	Pseudomonas sp. GM102	59.0	Contig	Oak Ridge National Lab
GCA_000282455	Cluster_4	Pseudomonas sp. GM74	60.1	Contig	Oak Ridge National Lab
GCA_000282355	Cluster_4	Pseudomonas sp. GM49	59.6	Contig	Oak Ridge National Lab
GCA_000282335	Cluster_4	Pseudomonas sp. GM48	59.4	Contig	Oak Ridge National Lab
GCA_000316175	Cluster_4	Pseudomonas sp. UW4	60.1	Complete Genome	University of Waterloo
GCA_000282295	Cluster_4	Pseudomonas sp. GM33	60.1	Contig	Oak Ridge National Lab
GCA_000282475	Cluster_5	Pseudomonas sp. GM78	60.2	Contig	Oak Ridge National Lab
GCA_000729805	Cluster_5	Pseudomonas putida 1	59.7	Contig	University of Groningen
GCA_000408945	Cluster_5	Pseudomonas sp. G5(2012)	59.3	Contig	Gwangju Institution of Science and Technology
GCA_000282155	Cluster_6	Pseudomonas sp. GM16	59.1	Contig	Oak Ridge National Lab
GCA_000783395	Cluster_6	Pseudomonas chlororaphis 1	59.2	Contig	University of Massachusetts Dartmouth
GCA_000276585	Cluster_6	Pseudomonas fluorescens NZ011	58.5	Contig	The Sainsbury Laboratory
GCA_000282235	Cluster_6	Pseudomonas sp. GM24	59.1	Contig	Oak Ridge National Lab
GCA_000631985	Cluster_7	Pseudomonas sp. RIT288	60.0	Contig	Monash University Malaysia
GCA_000633255	Cluster_7	Pseudomonas sp. H1h	60.3	Contig	University of Malaya
GCA_000514195	Cluster_7	Pseudomonas sp. URIL14HWK12:I6	60.0	Scaffold	DOE Joint Genome Institute
GCA_000282275	Cluster_7	Pseudomonas sp. GM30	60.3	Contig	Oak Ridge National Lab
GCA_000292795	Cluster_7	Pseudomonas fluorescens R124	60.3	Chromosome	Northern Kentucky University
GCA_000012445	Cluster_8	Pseudomonas fluorescens Pf0-1	60.5	Complete Genome	DOE Joint Genome Institute
GCA_000282255	Cluster_8	Pseudomonas sp. GM25	60.9	Contig	Oak Ridge National Lab
GCA_000512275	Cluster_9	Pseudomonas moraviensis R28	-	Chromosome	University of Idaho
GCA_000745605	Cluster_9	Pseudomonas sp. PTA1	59.7	Scaffold	DOE Joint Genome Institute
GCA_000257605	Cluster_9	Pseudomonas sp. R62	60.0	Scaffold	Botanical Institute
GCA_000785375	Cluster_10	Pseudomonas brassicacearum PP1_210F	60.9	Scaffold	CNRS - UPR2355
GCA_000498415	Cluster_10	Pseudomonas fluorescens S12	60.7	Contig	University of Malaya
GCA_000285615	Cluster_10	Pseudomonas fluorescens Wood1R	60.8	Contig	The Ohio State Univeristy
GCA_000194805	Cluster_10	Pseudomonas brassicacearum subsp. brassicacearum NFM421	60.8	Complete Genome	CEA/DSV/IBeB/SBVME/LEMIRE
GCA_000263695	Cluster_10	Pseudomonas fluorescens Q8r1-96	61.0	Chromosome	USDA - Agricultural Research Service, USA
GCA_000416195	Cluster_10	Pseudomonas sp. CFII68	60.8	Contig	Boise State University
GCA_000514275	Cluster_10	Pseudomonas sp. URIL14HWK12:I7	60.7	Scaffold	DOE Joint Genome Institute
GCA_000510785	Cluster_10	Pseudomonas brassicacearum 51MFCVI2.1	61.0	Scaffold	JGI
GCA_000237065	Cluster_10	Pseudomonas fluorescens F113	60.8	Complete Genome	Universidad Aut√≥noma de Madrid
GCA_000774145	Cluster_11	Pseudomonas mediterranea CFBP 5447	61.2	Scaffold	Parco Scientifico e tecnologico della Sicilia s.c.p.a.
GCA_000522485	Cluster_11	Pseudomonas corrugata CFBP 5454	60.5	Contig	Parco Scientifico e tecnologico della Sicilia s.c.p.a.
GCA_000285955	Cluster_12	Pseudomonas fluorescens Wayne1 1	63.3	Contig	The Ohio State University

GCA_000012265	Cluster_12	Pseudomonas protegens Pf-5	63.3	Complete Genome	TIGR
GCA_000397205	Cluster_12	Pseudomonas protegens CHA0	63.4	Complete Genome	Georg-August-University Goettingen, Genomic and Applied Microbiology, Goettingen Genomics Laboratory
GCA_000285355	Cluster_12	Pseudomonas fluorescens Wayne1	63.4	Contig	The Ohio State University
GCA_000698865	Cluster_13	Pseudomonas chlororaphis	62.6	Complete Genome	University of Manitoba
GCA_000282175	Cluster_13	Pseudomonas sp. GM17	62.8	Contig	Oak Ridge National Lab
GCA_000506385	Cluster_13	Pseudomonas chlororaphis subsp. aurantiaca PB-St2	63.2	Contig	University of Tuebingen
GCA_000512485	Cluster_13	Pseudomonas chlororaphis YL-1	63.1	Scaffold	Jiangsu Institute of Plant Protection
GCA_000237045	Cluster_13	Pseudomonas chlororaphis subsp. chlororaphis GP72	63.1	Contig	Shanghai Jiao Tong University
GCA_000761195	Cluster_13	Pseudomonas chlororaphis subsp. aurantiaca	62.8	Complete Genome	Shanghai Normal University
GCA_000264555	Cluster_13	Pseudomonas chlororaphis O6	62.9	Chromosome	U.S. Department of Agriculture, Agricultural Research Service
GCA_000597925	Cluster_13	Pseudomonas chlororaphis HT66	62.6	Contig	Shanghai JiaoTong University
GCA_000281915	Cluster_13	Pseudomonas chlororaphis subsp. aureofaciens 30-84	62.9	Chromosome	USDA - Agricultural Research Service, USA
GCA_000465595	Cluster_14	Pseudomonas fluorescens EGD-AQ6	60.5	Contig	National Environmental Engineering Research Institute-CSIR
GCA_000510895	Cluster_14	Pseudomonas sp. FH1	60.1	Contig	University of Exeter
GCA_000698265	Cluster_14	Pseudomonas simiae	60.3	Chromosome with gaps	Utrecht University, Utrecht, NETHERLANDS
GCA_000785125	Cluster_14	Pseudomonas simiae 1	60.3	Scaffold	Agriculture and Agri-Food Canada
GCA_000275925	Cluster_14	Pseudomonas fluorescens NZ052	60.1	Contig	The Sainsbury Laboratory
GCA_000257625	Cluster_14	Pseudomonas sp. R81	60.5	Scaffold	Botanical Institute
GCA_000411675	Cluster_14	Pseudomonas fluorescens LMG 5329	60.5	Contig	Centre of Microbial and Plant Genetics (CMPG), University of Leuven
GCA_000503215	Cluster_14	Pseudomonas sp. 2-92(2010)	60.4	Scaffold	Agriculture and Agri-Food Canada
GCA_000276565	Cluster_15	Pseudomonas tolaasii PMS117	60.3	Contig	University of Exeter
GCA_000316215	Cluster_15	Pseudomonas tolaasii 6264	61.0	Contig	Chungbuk National University
GCA_000511155	Cluster_16	Pseudomonas fluorescens FH5	60.0	Contig	University of Exeter
GCA_000698295	Cluster_16	Pseudomonas sp. WCS374	60.0	Chromosome with gaps	Utrecht University, Utrecht, NETHERLANDS
GCA_000262325	Cluster_16	Pseudomonas fluorescens A506	59.9	Complete Genome	USDA-ARS
GCA_000263675	Cluster_16	Pseudomonas fluorescens SS101	60.0	Chromosome	USDA - Agricultural Research Service, USA
GCA_000416175	Cluster_16	Pseudomonas sp. CF150	59.8	Contig	Boise State University
GCA_000416255	Cluster_16	Pseudomonas sp. CFT9	59.8	Contig	Boise State University
GCA_000785395	Cluster_16	Pseudomonas fluorescens PA4C2	60.2	Scaffold	CNRS - UPR2355
GCA_000744215	Cluster_16	Pseudomonas sp. Eur1 9.41	60.0	Contig	DOE Joint Genome Institute
GCA_000737955	Cluster_17	Pseudomonas sp. BRG-100	59.6	Contig	Agriculture and Agri-Food Canada
GCA_000263715	Cluster_17	Pseudomonas synxantha BG33R	59.7	Chromosome	USDA - Agricultural Research Service, USA
GCA_000336465	Cluster_18	Pseudomonas poae RE1-1-14	60.8	Complete Genome	Graz University of Technology
GCA_000334015	Cluster_18	Pseudomonas fluorescens BRIP34879	60.9	Scaffold	CSIRO
GCA_000313235	Cluster_19	Pseudomonas sp. PAMC 26793	60.6	Contig	Korea Polar Research Institute
GCA_000242655	Cluster_19	Pseudomonas sp. PAMC 25886	61.2	Contig	Korea Polar Research Institute
GCA_000297195	Cluster_19	Pseudomonas fluorescens BBc6R8	61.0	Contig	Institut National de la Recherche Agronomique
GCA_000278565	Cluster_19	Pseudomonas sp. Ag1	60.5	Contig	New Mexico State University
GCA_000510915	Cluster_20	Pseudomonas sp. FH4	60.1	Contig	University of Exeter
GCA_000708695	Cluster_20	Pseudomonas fluorescens	60.5	Contig	Vrije Universiteit Brussel
GCA_000251185	Cluster_21	Pseudomonas fuscovaginae UPB0736	61.5	Scaffold	University of Exeter
GCA_000467065	Cluster_21	Pseudomonas fuscovaginae ICMP 5940	61.2	Contig	Charles Sturt University
GCA_000467005	Cluster_21	Pseudomonas fuscovaginae DAR 77795	61.3	Contig	Charles Sturt University
GCA_000280575	Cluster_21	Pseudomonas fuscovaginae CB98818	61.4	Contig	Zhejiang University
GCA_000250595	Cluster_22	Pseudomonas fragi A22	58.6	Scaffold	BGI
GCA_000346225	Cluster_22	Pseudomonas sp. Lz4W	58.7	Contig	Centre for Cellular and Molecular Biology
GCA_000331385	Cluster_23	Pseudomonas syringae pv. syringae B64	59.0	Chromosome	Institute of Plant Biology, University of Zurich
GCA_000452805	Cluster_23	Pseudomonas syringae CC1458	59.1	Contig	University of Arizona
GCA_000452925	Cluster_23	Pseudomonas syringae CC94	59.3	Contig	University of Arizona

GCA_000597765	Cluster_23	Pseudomonas syringae DSM 10604	59.0	Contig	Institute of Microbial Technology
GCA_000282735	Cluster_23	Pseudomonas syringae pv. panici str. LMG 2367	59.0	Contig	Zhejiang University
GCA_000177515	Cluster_23	Pseudomonas syringae pv. syringae 642	59.2	Contig	Virginia Tech
GCA_000412165	Cluster_23	Pseudomonas syringae pv. syringae SM	59.0	Chromosome	Institute of Plant Biology, University of Zurich
GCA_000145825	Cluster_23	Pseudomonas syringae Cit 7	59.4	Scaffold	University of North Carolina at Chapel Hill
GCA_000334055	Cluster_23	Pseudomonas syringae BRIP34881	58.9	Scaffold	CSIRO
GCA_000800685	Cluster_23	Pseudomonas syringae 4	59.2	Contig	BIG
GCA_000452585	Cluster_23	Pseudomonas syringae CC457	59.1	Contig	University of Arizona
GCA_000452605	Cluster_23	Pseudomonas syringae CC440	59.1	Contig	University of Arizona
GCA_000145805	Cluster_23	Pseudomonas syringae pv. pisi str. 1704B	58.7	Scaffold	University of North Carolina at Chapel Hill
GCA_000498595	Cluster_23	Pseudomonas syringae pv. atrofaciens str. DSM 50255	59.2	Contig	University of Arizona
GCA_000452725	Cluster_23	Pseudomonas syringae CC1543	59.2	Contig	University of Arizona
GCA_000163315	Cluster_23	Pseudomonas syringae pv. syringae FF5	59.0	Scaffold	The Sainsbury Laboratory
GCA_000737225	Cluster_23	Pseudomonas syringae 1	59.3	Contig	University of Arizona
GCA_000452445	Cluster_23	Pseudomonas syringae pv. pisi str. PP1	58.8	Contig	University of Arizona
GCA_000585725	Cluster_23	Pseudomonas syringae pv. syringae str. B301D-R	59.2	Contig	Institute of Plant Biology, University of Zurich
GCA_000334035	Cluster_23	Pseudomonas syringae BRIP34876	58.9	Scaffold	CSIRO
GCA_000302815	Cluster_23	Pseudomonas syringae pv. avellanae str. ISPaVe037	59.2	Scaffold	University of Toronto Centre for the Analysis of Genome Evolution and Function
GCA_000452525	Cluster_23	Pseudomonas syringae USA011	59.2	Contig	University of Arizona
GCA_000012245	Cluster_23	Pseudomonas syringae pv. syringae B728a	59.2	Complete Genome	DOE Joint Genome Institute
GCA_000710085	Cluster_23	Pseudomonas syringae pv. atrofaciens LMG 5095	58.9	Contig	Chungbuk National University
GCA_000145905	Cluster_23	Pseudomonas syringae pv. aptata str. DSM 50252	59.1	Scaffold	University of North Carolina at Chapel Hill
GCA_000333995	Cluster_23	Pseudomonas syringae BRIP39023	59.2	Scaffold	CSIRO
GCA_000507185	Cluster_23	Pseudomonas syringae KCTC 12500	59.0	Scaffold	KRIBB
GCA_000738515	Cluster_23	Pseudomonas syringae pv. syringae	59.2	Scaffold	University of Exeter
GCA_000145925	Cluster_23	Pseudomonas syringae pv. aceris str. M302273PT	59.1	Scaffold	University of North Carolina at Chapel Hill
GCA_000145785	Cluster_23	Pseudomonas syringae pv. japonica str. M301072PT	58.9	Scaffold	University of North Carolina at Chapel Hill
GCA_000302795	Cluster_23	Pseudomonas syringae pv. avellanae str. ISPaVe013	59.1	Scaffold	University of Toronto Centre for the Analysis of Genome Evolution and Function
GCA_000452465	Cluster_23	Pseudomonas syringae pv. syringae 1212	59.1	Contig	University of Arizona
GCA_000452565	Cluster_23	Pseudomonas syringae UB303	59.2	Contig	University of Arizona
GCA_000146005	Cluster_24	Pseudomonas syringae pv. lachrymans str. M301315	56.9	Scaffold	University of North Carolina at Chapel Hill
GCA_000732035	Cluster_24	Pseudomonas savastanoi pv. savastanoi	57.9	Contig	International Centre for Genetic Engineering and Biotechnology
GCA_000751155	Cluster_24	Pseudomonas savastanoi pv. savastanoi 1	57.9	Contig	ICGEB
GCA_000012205	Cluster_24	Pseudomonas syringae pv. phaseolicola 1448A	57.9	Complete Genome	Cornell University
GCA_000145945	Cluster_24	Pseudomonas syringae pv. tabaci ATCC 11528	58.1	Scaffold	University of North Carolina at Chapel Hill
GCA_000145765	Cluster_24	Pseudomonas syringae pv. mori str. 301020	57.8	Scaffold	University of North Carolina at Chapel Hill
GCA_000187065	Cluster_24	Pseudomonas syringae pv. glycinea str. race 4	58.0	Contig	University of Illinois at Urbana & Champaign
GCA_000187045	Cluster_24	Pseudomonas syringae pv. glycinea str. B076	57.8	Contig	University of Illinois at Urbana & Champaign
GCA_000159835	Cluster_24	Pseudomonas syringae pv. tabaci str. ATCC 11528	58.0	Scaffold	The Sainsbury Laboratory
GCA_000275945	Cluster_24	Pseudomonas syringae pv. tabaci str. 6605	58.0	Contig	University of Exeter
GCA_000163255	Cluster_24	Pseudomonas syringae pv. aesculi str. NCPPB 3681	58.3	Scaffold	The Sainsbury Laboratory
GCA_000145685	Cluster_24	Pseudomonas syringae pv. aesculi str. 0893_23	58.3	Scaffold	University of North Carolina at Chapel Hill
GCA_000143005	Cluster_24	Pseudomonas syringae pv. glycinea str. race 4 1	58.0	Scaffold	University of North Carolina at Chapel Hill
GCA_000164015	Cluster_24	Pseudomonas savastanoi pv. savastanoi NCPPB 3335	57.9	Chromosome	University of Wisconsin-Madison
GCA_000225805	Cluster_24	Pseudomonas syringae pv. phaseolicola 1644R	58.0	Scaffold	University of North Carolina
GCA_000163275	Cluster_24	Pseudomonas syringae pv. aesculi str. 2250	58.3	Scaffold	The Sainsbury Laboratory
GCA_000245475	Cluster_25	Pseudomonas syringae pv. actinidiae CH2010-6	58.5	Scaffold	University of Exeter
GCA_000344415	Cluster_25	Pseudomonas syringae ICMP 18806	58.7	Contig	University of Otago
GCA_000416465	Cluster_25	Pseudomonas syringae pv. theae ICMP 3923	58.6	Contig	The New Zealand Institute for Plant & Food Research Limited

GCA_000344515	Cluster_25	Pseudomonas syringae pv. actinidiae ICMP 19455	58.5	Contig	University of Otago
GCA_000416785	Cluster_25	Pseudomonas syringae pv. actinidiae ICMP 18883	58.7	Contig	The New Zealand Institute for Plant & Food Research Limited
GCA_000344535	Cluster_25	Pseudomonas syringae pv. actinidiae ICMP 18807	58.8	Contig	University of Otago
GCA_000145885	Cluster_25	Pseudomonas syringae pv. lachrymans str. M302278PT	58.6	Scaffold	University of North Carolina at Chapel Hill
GCA_000416585	Cluster_25	Pseudomonas syringae pv. actinidiae ICMP 19101	58.4	Contig	The New Zealand Institute for Plant & Food Research Limited
GCA_000344335	Cluster_25	Pseudomonas syringae pv. actinidiae ICMP 9853	58.8	Contig	University of Otago
GCA_000245415	Cluster_25	Pseudomonas syringae pv. actinidiae CFBP 7286	58.5	Scaffold	University of Exeter
GCA_000344435	Cluster_25	Pseudomonas syringae pv. actinidiae TP1	58.5	Contig	University of Otago
GCA_000145745	Cluster_25	Pseudomonas syringae pv. morsprunorum str. M302280PT	58.6	Scaffold	University of North Carolina at Chapel Hill
GCA_000658965	Cluster_25	Pseudomonas syringae pv. actinidiae ICMP 9617	58.0	Chromosome	The New Zealand Institute for Plant & Food Research Limited
GCA_000416505	Cluster_25	Pseudomonas syringae pv. actinidiae ICMP 19073	58.8	Contig	The New Zealand Institute for Plant & Food Research Limited
GCA_000007805	Cluster_25	Pseudomonas syringae pv. tomato str. DC3000	58.3	Complete Genome	TIGR
GCA_000416545	Cluster_25	Pseudomonas syringae pv. actinidiae ICMP 19098	58.7	Contig	The New Zealand Institute for Plant & Food Research Limited
GCA_000416945	Cluster_25	Pseudomonas syringae pv. actinidiae ICMP 18801	58.6	Contig	The New Zealand Institute for Plant & Food Research Limited
GCA_000452905	Cluster_25	Pseudomonas syringae CC1544	59.1	Contig	University of Arizona
GCA_000416605	Cluster_25	Pseudomonas syringae pv. actinidiae ICMP 19079	58.1	Contig	The New Zealand Institute for Plant & Food Research Limited
GCA_000172895	Cluster_25	Pseudomonas syringae pv. tomato T1	58.6	Contig	The Sainsbury Laboratory
GCA_000344395	Cluster_25	Pseudomonas syringae pv. actinidiae ICMP 18804	58.7	Contig	University of Otago
GCA_000452625	Cluster_25	Pseudomonas syringae CC1630	58.5	Contig	University of Arizona
GCA_000416865	Cluster_25	Pseudomonas syringae pv. actinidiae ICMP 19104	58.8	Contig	The New Zealand Institute for Plant & Food Research Limited
GCA_000416845	Cluster_25	Pseudomonas syringae pv. actinidiae ICMP 19102	58.8	Contig	The New Zealand Institute for Plant & Food Research Limited
GCA_000233815	Cluster_25	Pseudomonas syringae pv. actinidiae str. CRAFRU8.43	58.5	Contig	Universit√† di Udine
GCA_000416765	Cluster_25	Pseudomonas syringae pv. actinidiae ICMP 19095	58.7	Contig	The New Zealand Institute for Plant & Food Research Limited
GCA_000444135	Cluster_25	Pseudomonas avellanae BPIC 631 1	58.5	Contig	CRA
GCA_000416725	Cluster_25	Pseudomonas syringae pv. actinidiae ICMP 19097	58.6	Contig	The New Zealand Institute for Plant & Food Research Limited
GCA_000177495	Cluster_25	Pseudomonas syringae pv. tomato NCPPB 1108	58.7	Contig	Virginia Tech
GCA_000409185	Cluster_25	Pseudomonas syringae	57.2	Scaffold	University of Exeter
GCA_000452845	Cluster_25	Pseudomonas syringae CC1416	59.0	Contig	University of Arizona
GCA_000245395	Cluster_25	Pseudomonas syringae pv. theae NCPPB 2598 1	58.5	Scaffold	University of Exeter
GCA_000177455	Cluster_25	Pseudomonas syringae pv. tomato K40	58.6	Contig	Virginia Tech
GCA_000416925	Cluster_25	Pseudomonas syringae pv. actinidiae ICMP 18886	58.4	Contig	Plant & Food Research
GCA_000233835	Cluster_25	Pseudomonas syringae pv. actinidiae str. NCPPB 3739	58.8	Contig	Universit√† di Udine
GCA_000416625	Cluster_25	Pseudomonas syringae pv. actinidiae ICMP 18807 1	58.2	Contig	The New Zealand Institute for Plant & Food Research Limited
GCA_000416525	Cluster_25	Pseudomonas syringae pv. actinidiae ICMP 19103	58.8	Contig	The New Zealand Institute for Plant & Food Research Limited
GCA_000416645	Cluster_25	Pseudomonas syringae pv. actinidiae ICMP 19096	58.4	Contig	The New Zealand Institute for Plant & Food Research Limited
GCA_000342185	Cluster_25	Pseudomonas syringae pv. actinidiae ICMP 18744	58.5	Contig	University of Otago
GCA_000416885	Cluster_25	Pseudomonas syringae pv. actinidiae ICMP 19072	58.8	Contig	Plant & Food Research
GCA_000344475	Cluster_25	Pseudomonas syringae pv. actinidiae str. Shaanxi_M228	58.4	Contig	University of Otago
GCA_000302915	Cluster_25	Pseudomonas avellanae BPIC 631	58.7	Scaffold	University of Toronto Centre for the Analysis of Genome Evolution and Function
GCA_000416745	Cluster_25	Pseudomonas syringae pv. actinidiae ICMP 19094	58.7	Contig	The New Zealand Institute for Plant & Food Research Limited
GCA_000177475	Cluster_25	Pseudomonas syringae pv. tomato Max13	58.7	Contig	Virginia Tech
GCA_000452545	Cluster_25	Pseudomonas syringae USA007	58.9	Contig	University of Arizona
GCA_000444115	Cluster_25	Pseudomonas syringae pv. theae NCPPB 2598	58.6	Contig	CRA
GCA_000416805	Cluster_25	Pseudomonas syringae pv. actinidiae ICMP 19099	58.7	Contig	The New Zealand Institute for Plant & Food Research Limited
GCA_000245455	Cluster_25	Pseudomonas syringae pv. actinidiae PA459	58.4	Scaffold	University of Exeter
GCA_000452685	Cluster_25	Pseudomonas syringae CC1559	58.9	Contig	University of Arizona
GCA_000344375	Cluster_25	Pseudomonas syringae pv. actinidiae ICMP 18800	58.5	Contig	University of Otago
GCA_000344355	Cluster_25	Pseudomonas syringae pv. actinidiae ICMP 18708	58.5	Contig	University of Otago
GCA_000416485	Cluster_25	Pseudomonas syringae pv. actinidiae ICMP 19071	58.8	Contig	The New Zealand Institute for Plant & Food Research Limited

GCA_000344455	Cluster_25	Pseudomonas syringae pv. actinidiae TP6-1	58.5	Contig	University of Otago
GCA_000344555	Cluster_25	Pseudomonas syringae pv. actinidiae ICMP 19439	58.5	Contig	University of Otago
GCA_000416665	Cluster_25	Pseudomonas syringae pv. actinidiae ICMP 9855	58.8	Contig	The New Zealand Institute for Plant & Food Research Limited
GCA_000344495	Cluster_25	Pseudomonas syringae pv. actinidiae str. Shaanxi_M7	58.4	Contig	University of Otago
GCA_000233795	Cluster_25	Pseudomonas syringae pv. actinidiae str. NCPPB 3871	58.8	Contig	Universitv† di Udine
GCA_000648735	Cluster_25	Pseudomonas syringae pv. actinidiae ICMP 18884	58.3	Chromosome	The New Zealand Institute for Plant & Food Research
GCA_000416825	Cluster_25	Pseudomonas syringae pv. actinidiae ICMP 19100	58.7	Contig	The New Zealand Institute for Plant & Food Research Limited
GCA_000441975	Cluster_25	Pseudomonas avellanae CRAFRUec1	58.9	Contig	CRA
GCA_000416705	Cluster_25	Pseudomonas syringae pv. actinidiae ICMP 19068	58.7	Contig	The New Zealand Institute for Plant & Food Research Limited
GCA_000416685	Cluster_25	Pseudomonas syringae pv. actinidiae ICMP 19070	58.6	Contig	The New Zealand Institute for Plant & Food Research Limited
GCA_000145865	Cluster_25	Pseudomonas syringae pv. actinidiae str. M303091	58.8	Scaffold	University of North Carolina at Chapel Hill
GCA_000245435	Cluster_25	Pseudomonas syringae pv. actinidiae KW41	58.8	Scaffold	University of Exeter
GCA_000452705	Cluster_26	Pseudomonas syringae CC1557	58.6	Complete Genome	University of Arizona
GCA_000452785	Cluster_26	Pseudomonas syringae CC1466	58.5	Contig	University of Arizona
GCA_000452665	Cluster_26	Pseudomonas syringae CC1583	59.0	Contig	University of Arizona
GCA_000452645	Cluster_27	Pseudomonas syringae CC1629	57.7	Contig	University of Arizona
GCA_000452765	Cluster_27	Pseudomonas syringae CC1513	57.9	Contig	University of Arizona
GCA_000773135	Cluster_27	Pseudomonas coronafaciens	57.7	Contig	Chungbuk National University
GCA_000156995	Cluster_27	Pseudomonas syringae pv. oryzae str. 1_6	57.9	Scaffold	University of North Carolina at Chapel Hill
GCA_000452505	Cluster_28	Pseudomonas viridiflava CC1582	59.3	Scaffold	University of Arizona
GCA_000307715	Cluster_28	Pseudomonas viridiflava UASWS0038	59.3	Contig	University of Applied Science of Western Switzerland//Geneva
GCA_000452485	Cluster_28	Pseudomonas viridiflava TA043	59.3	Scaffold	University of Arizona
GCA_000452745	Cluster_29	Pseudomonas syringae CC1524	59.1	Contig	University of Arizona
GCA_000452825	Cluster_29	Pseudomonas syringae CC1417	59.3	Contig	University of Arizona
GCA_000737235	Cluster_30	Pseudomonas syringae 2	58.4	Contig	University of Arizona
GCA_000416235	Cluster_30	Pseudomonas sp. CFII64	58.9	Contig	Boise State University
GCA_000708715	Cluster_31	Pseudomonas putida W15Oct28	62.7	Contig	Vrije Universiteit Brussel
GCA_000498395	Cluster_31	Pseudomonas putida S13.1.2	62.3	Contig	UNIVERSITY OF MALAYA
GCA_000410575	Cluster_31	Pseudomonas putida H8234	61.6	Complete Genome	Universidad de Huelva
GCA_000412675	Cluster_31	Pseudomonas putida NBRC 14164	62.3	Complete Genome	National Institute of Technology and Evaluation
GCA_000292285	Cluster_31	Pseudomonas sp. S13.1.2	62.0	Contig	University of Malaya
GCA_000507325	Cluster_31	Pseudomonas putida OUS82	61.8	Contig	SCELSE
GCA_000478865	Cluster_31	Pseudomonas putida SJ3	61.7	Contig	Kyungpook national university
GCA_000514295	Cluster_32	Pseudomonas sp. LAIL14HWK12:I6	62.1	Scaffold	DOE Joint Genome Institute
GCA_000514315	Cluster_32	Pseudomonas sp. LAIL14HWK12:I9	62.1	Scaffold	DOE Joint Genome Institute
GCA_000621245	Cluster_32	Pseudomonas monteilii NBRC 103158 = DSM 14164	61.5	Scaffold	DOE Joint Genome Institute
GCA_000483105	Cluster_32	Pseudomonas sp. URIL14HWK12:I4	62.0	Scaffold	DOE Joint Genome Institute
GCA_000514355	Cluster_32	Pseudomonas sp. LAMO17WK12:I4	62.2	Scaffold	DOE Joint Genome Institute
GCA_000514335	Cluster_32	Pseudomonas sp. LAIL14HWK12:I12	62.2	Scaffold	DOE Joint Genome Institute
GCA_000285395	Cluster_32	Pseudomonas putida B001	62.1	Contig	Chonnam National University
GCA_000730605	Cluster_32	Pseudomonas monteilii NBRC 103158 = DSM 14164 1	61.5	Contig	National Institute of Technology and Evaluation
GCA_000262005	Cluster_32	Pseudomonas monteilii QM	61.8	Contig	School of Environmental Science and Technology, Dalian University of Technology
GCA_000527055	Cluster_32	Pseudomonas sp. LAIL14HWK12:I5	62.1	Contig	DOE Joint Genome Institute
GCA_000495455	Cluster_33	Pseudomonas putida S12 1	61.1	Contig	RWTH Aachen University
GCA_000633915	Cluster_33	Pseudomonas monteilii	61.4	Scaffold	85303
GCA_000264665	Cluster_33	Pseudomonas putida ND6	61.7	Complete Genome	Department of Microbiology of Nankai University
GCA_000183645	Cluster_33	Pseudomonas putida BIRD-1	61.7	Complete Genome	Estacion Experimental del Zaidin, CSIC
GCA_000787655	Cluster_33	Pseudomonas putida 3	61.9	Contig	National Institute of Technology and Evaluation
GCA_000226475	Cluster_33	Pseudomonas putida str. Idaho	61.6	Contig	Shanghai Jiao Tong University

GCA_000281215	Cluster_33	Pseudomonas putida DOT-T1E	61.4	Complete Genome	EEZ-CSIC
GCA_000016865	Cluster_33	Pseudomonas putida F1	61.9	Complete Genome	US DOE Joint Genome Institute
GCA_000271965	Cluster_33	Pseudomonas putida SJTE-1	62.3	Contig	Shanghai Jiao Tong University
GCA_000390005	Cluster_33	Pseudomonas putida LF54	61.3	Contig	University of Tsukuba
GCA_000007565	Cluster_33	Pseudomonas putida KT2440	61.5	Complete Genome	TIGR
GCA_000799625	Cluster_33	Pseudomonas putida 2	61.8	Scaffold	DOE Joint Genome Institute
GCA_000294445	Cluster_33	Pseudomonas putida LS46	61.7	Contig	University of Manitoba,
GCA_000367825	Cluster_33	Pseudomonas putida TRO1	61.4	Contig	Aalborg University
GCA_000226035	Cluster_33	Pseudomonas putida B6-2	61.6	Contig	State Key Laboratory of Microbial Metabolism and School of Life Sciences and Biotechnology, Shanghai Jiao Tong University
GCA_000287915	Cluster_33	Pseudomonas putida S12	61.5	Contig	Shanghai Jiao Tong University
GCA_000510325	Cluster_34	Pseudomonas monteilii SB3101	62.5	Complete Genome	Aalborg University
GCA_000325725	Cluster_34	Pseudomonas putida HB3267	62.6	Complete Genome	Universidad de Huelva
GCA_000219705	Cluster_34	Pseudomonas putida S16	62.3	Complete Genome	Shanghai Jiao Tong University
GCA_000691565	Cluster_34	Pseudomonas putida	62.5	Complete Genome	Nanjing Agricultural University
GCA_000511325	Cluster_34	Pseudomonas sp. FGI182	63.3	Complete Genome	DOE Joint Genome Institute
GCA_000764405	Cluster_34	Pseudomonas plecoglossicida	62.1	Contig	Yeungnam University
GCA_000510285	Cluster_34	Pseudomonas monteilii SB3078	62.5	Complete Genome	Aalborg University
GCA_000500605	Cluster_34	Pseudomonas taiwanensis SJ9	61.8	Contig	Kyungpook national university
GCA_000710785	Cluster_35	Pseudomonas putida T2-2	62.6	Contig	University of Malaya
GCA_000190455	Cluster_35	Pseudomonas sp. TJI-51	62.1	Contig	ICCBS
GCA_000763225	Cluster_36	Pseudomonas sp. H2	62.6	Contig	University of Idaho
GCA_000731675	Cluster_36	Pseudomonas sp. WCS358	62.7	Contig	Utrecht University, Utrecht, NETHERLANDS
GCA_000412715	Cluster_37	Pseudomonas putida NB2011	62.8	Scaffold	Zhejiang Wanli University
GCA_000730665	Cluster_37	Pseudomonas plecoglossicida NBRC 103162 = DSM 15088 1	63.0	Contig	National Institute of Technology and Evaluation
GCA_000688275	Cluster_37	Pseudomonas plecoglossicida NBRC 103162 = DSM 15088	63.0	Contig	DOE Joint Genome Institute
GCA_000494915	Cluster_38	Pseudomonas sp. VLB120	61.5	Complete Genome	Bielefeld University
GCA_000425785	Cluster_38	Pseudomonas taiwanensis DSM 21245	61.9	Scaffold	DOE Joint Genome Institute
GCA_000514235	Cluster_39	Pseudomonas sp. URMO17WK12:I11	61.9	Scaffold	DOE Joint Genome Institute
GCA_000730565	Cluster_39	Pseudomonas fulva NBRC 16637 = DSM 17717 1	61.8	Contig	National Institute of Technology and Evaluation
GCA_000621265	Cluster_39	Pseudomonas fulva NBRC 16637 = DSM 17717	61.7	Scaffold	DOE Joint Genome Institute
GCA_000730545	Cluster_39	Pseudomonas cremoricolorata NBRC 16634	61.7	Contig	National Institute of Technology and Evaluation
GCA_000425765	Cluster_40	Pseudomonas parafulva DSM 17004	62.5	Scaffold	DOE Joint Genome Institute
GCA_000730645	Cluster_40	Pseudomonas parafulva NBRC 16636	62.5	Contig	National Institute of Technology and Evaluation
GCA_000800255	Cluster_41	Pseudomonas parafulva	63.5	Complete Genome	China National Rice Research Institute
GCA_000467045	Cluster_41	Pseudomonas sp. ICMP 17674	63.4	Contig	Charles Sturt University
GCA_000696345	Cluster_42	Pseudomonas sp. P482	62.4	Contig	Intercollegiate Faculty of Biotechnology UG&MUG
GCA_000259195	Cluster_42	Pseudomonas sp. HYS	62.4	Scaffold	College of Life Sciences, Wuhan University
GCA_000733715	Cluster_43	Pseudomonas mendocina S5.2	62.4	Contig	University of Malaya
GCA_000204295	Cluster_43	Pseudomonas mendocina NK-01	62.5	Complete Genome	Department of Microbiology, College of Life Sciences, Nankai University,Tianjin 300071, China
GCA_000725105	Cluster_43	Pseudomonas mendocina	62.4	Contig	University of Malaya
GCA_000319815	Cluster_44	Pseudomonas alcaliphila 34	62.6	Contig	University of Florence
GCA_000465575	Cluster_44	Pseudomonas mendocina EGD-AQ5	62.7	Contig	National Environmental Engineering Research Institute-CSIR
GCA_000016565	Cluster_45	Pseudomonas mendocina ymp	64.7	Complete Genome	US DOE Joint Genome Institute
GCA_000287395	Cluster_45	Pseudomonas mendocina DLHK	64.7	Contig	The University of Hong Kong
GCA_000732445	Cluster_46	Pseudomonas oleovorans	64.9	Contig	85303
GCA_000798915	Cluster_46	Pseudomonas mendocina ZWU0006	64.8	Contig	University of Oregon
GCA_000514255	Cluster_47	Pseudomonas sp. URMO17WK12:I3	63.6	Scaffold	DOE Joint Genome Institute
GCA_000514215	Cluster_47	Pseudomonas sp. URMO17WK12:I4	63.4	Scaffold	DOE Joint Genome Institute
GCA_000213805	Cluster_47	Pseudomonas fulva 12-X	63.5	Complete Genome	US DOE Joint Genome Institute

GCA_000495915	Cluster_48	Pseudomonas chloritidismutans AW-1	62.5	Contig	Laboratory of Systems and Synthetic Biology
GCA_000267545	Cluster_48	Pseudomonas stutzeri CCUG 29243	62.7	Complete Genome	MICROGEN: Microbial Comparative Genomics
GCA_000341615	Cluster_48	Pseudomonas stutzeri NF13	63.0	Contig	University of Balearic Islands
GCA_000416345	Cluster_49	Pseudomonas stutzeri B1SMN1	63.4	Contig	University of Balearic Islands
GCA_000280555	Cluster_49	Pseudomonas stutzeri XLDN-R	63.9	Contig	State Key Laboratory of Microbial Metabolism and School of Life Sciences and Biotechnology, Shanghai Jiao Tong University
GCA_000195105	Cluster_49	Pseudomonas stutzeri DSM 4166	64.0	Complete Genome	Biotechnology Research Institute, Chinese Academy of Agricultural Sciences, Key Laboratory of Crop Biotechnology.
GCA_000282955	Cluster_49	Pseudomonas stutzeri T13	63.9	Contig	Harbin Institute of Technology
GCA_000474255	Cluster_50	Pseudomonas alcaligenes MRY13-0052	65.8	Contig	National Institute of Infectious Diseases, Japan
GCA_000455385	Cluster_50	Pseudomonas alcaligenes OT 69	66.0	Contig	University of Illinois at Chicago
GCA_000733615	Cluster_51	Pseudomonas nitroreducens	64.2	Contig	Georgia Institute of Technology
GCA_000733635	Cluster_51	Pseudomonas nitroreducens 1	64.2	Contig	Georgia Institute of Technology
GCA_000518065	Cluster_51	Pseudomonas nitroreducens HBP1	64.2	Contig	CIB-CSIC
GCA_000317185	Cluster_52	Pseudomonas sp. M1	67.2	Contig	University of Minho
GCA_000725445	Cluster_52	Pseudomonas sp. AAC	67.0	Scaffold	CSIRO
GCA_000792945	Cluster_53	Pseudomonas aeruginosa 188	66.4	Contig	AstraZeneca
GCA_000795735	Cluster_53	Pseudomonas aeruginosa 322	66.0	Contig	AstraZeneca
GCA_000795025	Cluster_53	Pseudomonas aeruginosa 286	66.4	Contig	AstraZeneca
GCA_000791445	Cluster_53	Pseudomonas aeruginosa 111	66.3	Contig	AstraZeneca
GCA_000520355	Cluster_53	Pseudomonas aeruginosa BWHPSA042	66.5	Scaffold	Broad Institute
GCA_000478485	Cluster_53	Pseudomonas sp. P179	65.8	Scaffold	Broad Institute
GCA_000786565	Cluster_53	Pseudomonas aeruginosa 10	66.2	Contig	University of Huddersfield
GCA_000484495	Cluster_53	Pseudomonas aeruginosa PAO1-VE2	66.6	Chromosome with gaps	Marshall University School of Medicine
GCA_000792545	Cluster_53	Pseudomonas aeruginosa 168	66.1	Contig	AstraZeneca
GCA_000506345	Cluster_53	Pseudomonas aeruginosa JD333	66.5	Contig	University of Ottawa
GCA_000796505	Cluster_53	Pseudomonas aeruginosa 369	66.5	Contig	AstraZeneca
GCA_000795345	Cluster_53	Pseudomonas aeruginosa 302	65.9	Contig	AstraZeneca
GCA_000793665	Cluster_53	Pseudomonas aeruginosa 221	66.5	Contig	AstraZeneca
GCA_000792965	Cluster_53	Pseudomonas aeruginosa 189	65.9	Contig	AstraZeneca
GCA_000790465	Cluster_53	Pseudomonas aeruginosa 151	66.3	Contig	AstraZeneca
GCA_000789835	Cluster_53	Pseudomonas aeruginosa 31	66.3	Contig	AstraZeneca
GCA_000506185	Cluster_53	Pseudomonas aeruginosa JD322	66.4	Contig	University of Ottawa
GCA_000247435	Cluster_53	Pseudomonas aeruginosa MPAO1/P1	66.5	Contig	Argonne National Laboratory
GCA_000223965	Cluster_53	Pseudomonas aeruginosa 213BR	66.1	Complete Genome	IBIS, Universite Laval
GCA_000789815	Cluster_53	Pseudomonas aeruginosa 30	66.4	Contig	AstraZeneca
GCA_000796425	Cluster_53	Pseudomonas aeruginosa 365	66.3	Contig	AstraZeneca
GCA_000789725	Cluster_53	Pseudomonas aeruginosa 25	66.0	Contig	AstraZeneca
GCA_000481725	Cluster_53	Pseudomonas aeruginosa S35004	66.1	Scaffold	Broad Institute
GCA_000791855	Cluster_53	Pseudomonas aeruginosa 132	66.0	Contig	AstraZeneca
GCA_000790485	Cluster_53	Pseudomonas aeruginosa 63	66.4	Contig	AstraZeneca
GCA_000793365	Cluster_53	Pseudomonas aeruginosa 209	66.5	Contig	AstraZeneca
GCA_000481305	Cluster_53	Pseudomonas aeruginosa BWHPSA020	66.3	Scaffold	Broad Institute
GCA_000790735	Cluster_53	Pseudomonas aeruginosa 76	66.0	Contig	AstraZeneca
GCA_000795985	Cluster_53	Pseudomonas aeruginosa 343	66.0	Contig	AstraZeneca
GCA_000629185	Cluster_53	Pseudomonas aeruginosa 3580	66.0	Scaffold	Broad Institute
GCA_000481585	Cluster_53	Pseudomonas aeruginosa BWHPSA006	66.2	Scaffold	Broad Institute
GCA_000796345	Cluster_53	Pseudomonas aeruginosa 361	65.8	Contig	AstraZeneca
GCA_000795145	Cluster_53	Pseudomonas aeruginosa 292	66.1	Contig	AstraZeneca
GCA_000412355	Cluster_53	Pseudomonas aeruginosa str. C 763	66.0	Contig	University of Strathclyde
GCA_000791025	Cluster_53	Pseudomonas aeruginosa 90	66.3	Contig	AstraZeneca

GCA_000757505	Cluster_53	Pseudomonas sp. YS-1p	66.5	Contig	Oklahoma State University
GCA_000629465	Cluster_53	Pseudomonas aeruginosa BWH056	66.0	Scaffold	Broad Institute
GCA_000792745	Cluster_53	Pseudomonas aeruginosa 178	66.4	Contig	AstraZeneca
GCA_000793745	Cluster_53	Pseudomonas aeruginosa 225	66.1	Contig	AstraZeneca
GCA_000481845	Cluster_53	Pseudomonas aeruginosa UDL	66.5	Scaffold	Broad Institute
GCA_000480435	Cluster_53	Pseudomonas aeruginosa C48	66.4	Scaffold	Broad Institute
GCA_000796365	Cluster_53	Pseudomonas aeruginosa 362	65.9	Contig	AstraZeneca
GCA_000793135	Cluster_53	Pseudomonas aeruginosa 198	65.7	Contig	AstraZeneca
GCA_000789545	Cluster_53	Pseudomonas aeruginosa 17	66.5	Contig	AstraZeneca
GCA_000795705	Cluster_53	Pseudomonas aeruginosa 320	66.1	Contig	AstraZeneca
GCA_000789605	Cluster_53	Pseudomonas aeruginosa 19	66.0	Contig	AstraZeneca
GCA_000792005	Cluster_53	Pseudomonas aeruginosa 139	66.5	Contig	AstraZeneca
GCA_000790685	Cluster_53	Pseudomonas aeruginosa 73	66.2	Contig	AstraZeneca
GCA_000481925	Cluster_53	Pseudomonas aeruginosa CF18	66.3	Scaffold	Broad Institute
GCA_000629145	Cluster_53	Pseudomonas aeruginosa BWH029	66.4	Scaffold	Broad Institute
GCA_000795565	Cluster_53	Pseudomonas aeruginosa 313	66.0	Contig	AstraZeneca
GCA_000792365	Cluster_53	Pseudomonas aeruginosa 159	66.4	Contig	AstraZeneca
GCA_000520195	Cluster_53	Pseudomonas aeruginosa PS42	65.5	Scaffold	Broad Institute
GCA_000792085	Cluster_53	Pseudomonas aeruginosa 143	65.8	Contig	AstraZeneca
GCA_000168335	Cluster_53	Pseudomonas aeruginosa PACS2	66.3	Complete Genome	University of Washington
GCA_000791635	Cluster_53	Pseudomonas aeruginosa 121	66.4	Contig	AstraZeneca
GCA_000796245	Cluster_53	Pseudomonas aeruginosa 356	66.0	Contig	AstraZeneca
GCA_000794305	Cluster_53	Pseudomonas aeruginosa 251	66.3	Contig	AstraZeneca
GCA_000795865	Cluster_53	Pseudomonas aeruginosa 328	65.8	Contig	AstraZeneca
GCA_000481665	Cluster_53	Pseudomonas aeruginosa BWHPSA002	66.1	Scaffold	Broad Institute
GCA_000792145	Cluster_53	Pseudomonas aeruginosa 146	66.0	Contig	AstraZeneca
GCA_000629205	Cluster_53	Pseudomonas aeruginosa 3579	66.2	Scaffold	Broad Institute
GCA_000583975	Cluster_53	Pseudomonas aeruginosa LESlike1	66.4	Chromosome with gaps	IBIS, University Laval
GCA_000794085	Cluster_53	Pseudomonas aeruginosa 242	65.8	Contig	AstraZeneca
GCA_000790585	Cluster_53	Pseudomonas aeruginosa 68	66.0	Contig	AstraZeneca
GCA_000763245	Cluster_53	Pseudomonas aeruginosa 8	66.2	Contig	Instituto Oswaldo Cruz
GCA_000481105	Cluster_53	Pseudomonas aeruginosa BL02	66.1	Scaffold	Broad Institute
GCA_000794965	Cluster_53	Pseudomonas aeruginosa 283	66.5	Contig	AstraZeneca
GCA_000790035	Cluster_53	Pseudomonas aeruginosa 41	66.2	Contig	AstraZeneca
GCA_000791185	Cluster_53	Pseudomonas aeruginosa 98	66.0	Contig	AstraZeneca
GCA_000793885	Cluster_53	Pseudomonas aeruginosa 232	65.9	Contig	AstraZeneca
GCA_000480575	Cluster_53	Pseudomonas aeruginosa M8A.3	66.5	Scaffold	Broad Institute
GCA_000797085	Cluster_53	Pseudomonas aeruginosa 398	65.6	Contig	AstraZeneca
GCA_000793615	Cluster_53	Pseudomonas aeruginosa 219	66.2	Contig	AstraZeneca
GCA_000791155	Cluster_53	Pseudomonas aeruginosa 97	65.9	Contig	AstraZeneca
GCA_000795115	Cluster_53	Pseudomonas aeruginosa 291	65.9	Contig	AstraZeneca
GCA_000793105	Cluster_53	Pseudomonas aeruginosa 196	66.6	Contig	AstraZeneca
GCA_000647595	Cluster_53	Pseudomonas aeruginosa 152	66.1	Contig	Universidad Nacional Autonoma de Mexico
GCA_000583935	Cluster_53	Pseudomonas aeruginosa LES400	66.3	Chromosome with gaps	IBIS, University Laval
GCA_000520435	Cluster_53	Pseudomonas aeruginosa BWHPSA038	66.3	Scaffold	Broad Institute
GCA_000795535	Cluster_53	Pseudomonas aeruginosa 312	66.0	Contig	AstraZeneca
GCA_000258285	Cluster_53	Pseudomonas aeruginosa LCT-PA102	66.2	Contig	BGI
GCA_000295475	Cluster_53	Pseudomonas aeruginosa CIG1	66.0	Contig	University of Washington
GCA_000792565	Cluster_53	Pseudomonas aeruginosa 169	66.3	Contig	AstraZeneca

GCA_000412455	Cluster_53	Pseudomonas aeruginosa str. PA 17	66.5	Contig	University of Strathclyde
GCA_000791265	Cluster_53	Pseudomonas aeruginosa 102	66.3	Contig	AstraZeneca
GCA_000760505	Cluster_53	Pseudomonas aeruginosa 12	65.7	Contig	Center for Cellular and Molecular Biology(CCMB)
GCA_000480745	Cluster_53	Pseudomonas aeruginosa BL20	66.2	Scaffold	Broad Institute
GCA_000797125	Cluster_53	Pseudomonas aeruginosa 400	66.3	Contig	AstraZeneca
GCA_000297315	Cluster_53	Pseudomonas aeruginosa ATCC 700888	66.1	Contig	University of Washington
GCA_000790825	Cluster_53	Pseudomonas aeruginosa 80	66.0	Contig	AstraZeneca
GCA_000794025	Cluster_53	Pseudomonas aeruginosa 239	66.1	Contig	AstraZeneca
GCA_000506325	Cluster_53	Pseudomonas aeruginosa JD332	66.6	Contig	University of Ottawa
GCA_000793205	Cluster_53	Pseudomonas aeruginosa 201	66.4	Contig	AstraZeneca
GCA_000791685	Cluster_53	Pseudomonas aeruginosa 123	65.4	Contig	AstraZeneca
GCA_000291745	Cluster_53	Pseudomonas aeruginosa NCMG1179	66.0	Scaffold	National Center for Global Health and Medicine
GCA_000796865	Cluster_53	Pseudomonas aeruginosa 387	66.0	Contig	AstraZeneca
GCA_000795105	Cluster_53	Pseudomonas aeruginosa 290	66.0	Contig	AstraZeneca
GCA_000793585	Cluster_53	Pseudomonas aeruginosa 217	66.4	Contig	AstraZeneca
GCA_000794445	Cluster_53	Pseudomonas aeruginosa 257	66.1	Contig	AstraZeneca
GCA_000791305	Cluster_53	Pseudomonas aeruginosa 104	65.9	Contig	AstraZeneca
GCA_000792395	Cluster_53	Pseudomonas aeruginosa 161	66.1	Contig	AstraZeneca
GCA_000520275	Cluster_53	Pseudomonas aeruginosa BWHPSA046	66.1	Scaffold	Broad Institute
GCA_000792155	Cluster_53	Pseudomonas aeruginosa 147	66.4	Contig	AstraZeneca
GCA_000796045	Cluster_53	Pseudomonas aeruginosa 346	66.0	Contig	AstraZeneca
GCA_000794405	Cluster_53	Pseudomonas aeruginosa 255	65.8	Contig	AstraZeneca
GCA_000793395	Cluster_53	Pseudomonas aeruginosa 211	65.8	Contig	AstraZeneca
GCA_000284555	Cluster_53	Pseudomonas aeruginosa NCGM2.S1	66.1	Complete Genome	National Center for Global Health and Medicine
GCA_000709285	Cluster_53	Pseudomonas aeruginosa 1	66.5	Contig	University of Delhi
GCA_000792425	Cluster_53	Pseudomonas aeruginosa 162	66.4	Contig	AstraZeneca
GCA_000791525	Cluster_53	Pseudomonas aeruginosa 115	66.3	Contig	AstraZeneca
GCA_000796875	Cluster_53	Pseudomonas aeruginosa 388	66.5	Contig	AstraZeneca
GCA_000629045	Cluster_53	Pseudomonas aeruginosa BWH035	66.1	Scaffold	Broad Institute
GCA_000789485	Cluster_53	Pseudomonas aeruginosa 13	66.5	Contig	AstraZeneca
GCA_000506285	Cluster_53	Pseudomonas aeruginosa JD334	66.2	Contig	University of Ottawa
GCA_000794665	Cluster_53	Pseudomonas aeruginosa 268	66.1	Contig	AstraZeneca
GCA_000480475	Cluster_53	Pseudomonas aeruginosa C40	66.3	Scaffold	Broad Institute
GCA_000789805	Cluster_53	Pseudomonas aeruginosa 29	66.4	Contig	AstraZeneca
GCA_000790645	Cluster_53	Pseudomonas aeruginosa 71	66.5	Contig	AstraZeneca
GCA_000481225	Cluster_53	Pseudomonas aeruginosa BWHPSA024	66.1	Scaffold	Broad Institute
GCA_000520175	Cluster_53	Pseudomonas aeruginosa PS50	66.0	Scaffold	Broad Institute
GCA_000791035	Cluster_53	Pseudomonas aeruginosa 91	66.2	Contig	AstraZeneca
GCA_000792065	Cluster_53	Pseudomonas aeruginosa 142	65.8	Contig	AstraZeneca
GCA_000505885	Cluster_53	Pseudomonas aeruginosa JD303	66.6	Contig	University of Ottawa
GCA_000794245	Cluster_53	Pseudomonas aeruginosa 248	66.0	Contig	AstraZeneca
GCA_000795165	Cluster_53	Pseudomonas aeruginosa 293	66.5	Contig	AstraZeneca
GCA_000791495	Cluster_53	Pseudomonas aeruginosa 114	66.1	Contig	AstraZeneca
GCA_000583995	Cluster_53	Pseudomonas aeruginosa LESlike4	66.4	Chromosome with gaps	IBIS, University Laval
GCA_000481345	Cluster_53	Pseudomonas aeruginosa BWHPSA018	66.2	Scaffold	Broad Institute
GCA_000412475	Cluster_53	Pseudomonas aeruginosa str. J 1532	66.2	Contig	University of Strathclyde
GCA_000791885	Cluster_53	Pseudomonas aeruginosa 133	66.2	Contig	AstraZeneca
GCA_000481265	Cluster_53	Pseudomonas aeruginosa BWHPSA022	66.2	Scaffold	Broad Institute
GCA_000795285	Cluster_53	Pseudomonas aeruginosa 299	66.1	Contig	AstraZeneca

GCA_000152545	Cluster_53	Pseudomonas aeruginosa 2192	66.2	Scaffold	Broad Institute
GCA_000795185	Cluster_53	Pseudomonas aeruginosa 294	66.4	Contig	AstraZeneca
GCA_000789525	Cluster_53	Pseudomonas aeruginosa 15	66.0	Contig	AstraZeneca
GCA_000520395	Cluster_53	Pseudomonas aeruginosa BWHPSA040	66.4	Scaffold	Broad Institute
GCA_000795065	Cluster_53	Pseudomonas aeruginosa 288	66.0	Contig	AstraZeneca
GCA_000615525	Cluster_53	Pseudomonas aeruginosa JCM 6119	65.8	Contig	The University of Tokyo
GCA_000789635	Cluster_53	Pseudomonas aeruginosa 21	66.0	Contig	AstraZeneca
GCA_000792265	Cluster_53	Pseudomonas aeruginosa 154	65.7	Contig	AstraZeneca
GCA_000480375	Cluster_53	Pseudomonas aeruginosa CF77	65.8	Scaffold	Broad Institute
GCA_000524595	Cluster_53	Pseudomonas aeruginosa YL84	66.4	Complete Genome	UNIVERSITY OF MALAYA
GCA_000481245	Cluster_53	Pseudomonas aeruginosa BWHPSA023	66.1	Scaffold	Broad Institute
GCA_000791425	Cluster_53	Pseudomonas aeruginosa 110	66.1	Contig	AstraZeneca
GCA_000792705	Cluster_53	Pseudomonas aeruginosa 176	65.8	Contig	AstraZeneca
GCA_000795585	Cluster_53	Pseudomonas aeruginosa 314	65.7	Contig	AstraZeneca
GCA_000791605	Cluster_53	Pseudomonas aeruginosa 119	66.2	Contig	AstraZeneca
GCA_000797005	Cluster_53	Pseudomonas aeruginosa 394	66.0	Contig	AstraZeneca
GCA_000791725	Cluster_53	Pseudomonas aeruginosa 125	65.8	Contig	AstraZeneca
GCA_000647655	Cluster_53	Pseudomonas aeruginosa M10	66.9	Contig	Universidad Nacional Autonoma de Mexico
GCA_000796145	Cluster_53	Pseudomonas aeruginosa 351	66.3	Contig	AstraZeneca
GCA_000789685	Cluster_53	Pseudomonas aeruginosa 23	66.1	Contig	AstraZeneca
GCA_000481445	Cluster_53	Pseudomonas aeruginosa BWHPSA013	66.2	Scaffold	Broad Institute
GCA_000496455	Cluster_53	Pseudomonas aeruginosa DHS01	65.8	Contig	Hospital of Besancon - France
GCA_000629365	Cluster_53	Pseudomonas aeruginosa PS75	66.5	Scaffold	Broad Institute
GCA_000233495	Cluster_53	Pseudomonas sp. 2_1_26	66.4	Scaffold	Broad Institute
GCA_000215795	Cluster_53	Pseudomonas aeruginosa HB15	66.2	Contig	University of Minho
GCA_000793785	Cluster_53	Pseudomonas aeruginosa 227	65.9	Contig	AstraZeneca
GCA_000705175	Cluster_53	Pseudomonas aeruginosa C2773C	65.9	Contig	University of Texas at Austin
GCA_000794585	Cluster_53	Pseudomonas aeruginosa 264	65.9	Contig	AstraZeneca
GCA_000790245	Cluster_53	Pseudomonas aeruginosa 51	66.4	Contig	AstraZeneca
GCA_000796615	Cluster_53	Pseudomonas aeruginosa 375	66.6	Contig	AstraZeneca
GCA_000439875	Cluster_53	Pseudomonas aeruginosa LCT-PA41	66.2	Scaffold	BGI Research Institute
GCA_000480845	Cluster_53	Pseudomonas aeruginosa BL15	66.5	Scaffold	Broad Institute
GCA_000789975	Cluster_53	Pseudomonas aeruginosa 38	66.5	Contig	AstraZeneca
GCA_000480455	Cluster_53	Pseudomonas aeruginosa C41	66.2	Scaffold	Broad Institute
GCA_000791765	Cluster_53	Pseudomonas aeruginosa 127	66.5	Contig	AstraZeneca
GCA_000480685	Cluster_53	Pseudomonas aeruginosa BL23	65.7	Scaffold	Broad Institute
GCA_000793185	Cluster_53	Pseudomonas aeruginosa 200	66.4	Contig	AstraZeneca
GCA_000795725	Cluster_53	Pseudomonas aeruginosa 321	66.0	Contig	AstraZeneca
GCA_000796465	Cluster_53	Pseudomonas aeruginosa 367	66.3	Contig	AstraZeneca
GCA_000271365	Cluster_53	Pseudomonas aeruginosa DK2	66.3	Complete Genome	Technical University of Denmark
GCA_000796065	Cluster_53	Pseudomonas aeruginosa 347	66.5	Contig	AstraZeneca
GCA_000783275	Cluster_53	Pseudomonas aeruginosa 9	65.8	Scaffold	China CDC
GCA_000480725	Cluster_53	Pseudomonas aeruginosa BL21	66.1	Scaffold	Broad Institute
GCA_000647935	Cluster_53	Pseudomonas aeruginosa RB	66.6	Scaffold	Osaka University
GCA_000481125	Cluster_53	Pseudomonas aeruginosa BL01	66.4	Scaffold	Broad Institute
GCA_000629325	Cluster_53	Pseudomonas aeruginosa 3573	66.5	Scaffold	Broad Institute
GCA_000791225	Cluster_53	Pseudomonas aeruginosa 100	65.8	Contig	AstraZeneca
GCA_000399805	Cluster_53	Pseudomonas aeruginosa VRFPA02	66.0	Contig	SANKARA NETHRALAYA, VISION RESEARCH FOUNDATION
GCA_000791545	Cluster_53	Pseudomonas aeruginosa 116	65.9	Contig	AstraZeneca

GCA_000790835	Cluster_53	Pseudomonas aeruginosa 81	66.0	Contig	AstraZeneca
GCA_000629125	Cluster_53	Pseudomonas aeruginosa BWH030	66.4	Scaffold	Broad Institute
GCA_000791665	Cluster_53	Pseudomonas aeruginosa 122	65.8	Contig	AstraZeneca
GCA_000791145	Cluster_53	Pseudomonas aeruginosa 96	65.9	Contig	AstraZeneca
GCA_000629025	Cluster_53	Pseudomonas aeruginosa BWH036	66.2	Scaffold	Broad Institute
GCA_000796925	Cluster_53	Pseudomonas aeruginosa 390	66.6	Contig	AstraZeneca
GCA_000796545	Cluster_53	Pseudomonas aeruginosa 371	66.1	Contig	AstraZeneca
GCA_000215775	Cluster_53	Pseudomonas aeruginosa HB13	66.2	Contig	University of Minho
GCA_000794485	Cluster_53	Pseudomonas aeruginosa 259	66.0	Contig	AstraZeneca
GCA_000793445	Cluster_53	Pseudomonas aeruginosa 213	66.1	Contig	AstraZeneca
GCA_000790215	Cluster_53	Pseudomonas aeruginosa 50	65.9	Contig	AstraZeneca
GCA_000568215	Cluster_53	Pseudomonas aeruginosa SG17M	66.0	Contig	Karolinska Institute
GCA_000583895	Cluster_53	Pseudomonas aeruginosa LESlike5	66.4	Chromosome with gaps	IBIS, University Laval
GCA_000481625	Cluster_53	Pseudomonas aeruginosa BWHPSA004	66.5	Scaffold	Broad Institute
GCA_000478465	Cluster_53	Pseudomonas aeruginosa str. Stone 130	65.4	Scaffold	Broad Institute
GCA_000014625	Cluster_53	Pseudomonas aeruginosa UCBPP-PA14	66.3	Complete Genome	Massachusetts General Hospital
GCA_000795045	Cluster_53	Pseudomonas aeruginosa 287	66.0	Contig	AstraZeneca
GCA_000791405	Cluster_53	Pseudomonas aeruginosa 109	66.1	Contig	AstraZeneca
GCA_000412495	Cluster_53	Pseudomonas aeruginosa str. PA 17SCV	66.4	Contig	University of Strathclyde
GCA_000794605	Cluster_53	Pseudomonas aeruginosa 265	66.6	Contig	AstraZeneca
GCA_000531435	Cluster_53	Pseudomonas aeruginosa PA38182	64.9	Chromosome with gaps	BUGS
GCA_000793865	Cluster_53	Pseudomonas aeruginosa 231	66.5	Contig	AstraZeneca
GCA_000793255	Cluster_53	Pseudomonas aeruginosa 204	66.1	Contig	AstraZeneca
GCA_000794645	Cluster_53	Pseudomonas aeruginosa 267	65.9	Contig	AstraZeneca
GCA_000792045	Cluster_53	Pseudomonas aeruginosa 141	66.2	Contig	AstraZeneca
GCA_000792505	Cluster_53	Pseudomonas aeruginosa 166	66.3	Contig	AstraZeneca
GCA_000414035	Cluster_53	Pseudomonas aeruginosa RP73	66.5	Complete Genome	San Raffaele Scientific Institute, Division of Immunology, Transplantation and Infectious Diseases, Milano, Italy
GCA_000455505	Cluster_53	Pseudomonas aeruginosa B3-20M	66.2	Contig	DTU
GCA_000792885	Cluster_53	Pseudomonas aeruginosa 185	65.9	Contig	AstraZeneca
GCA_000484545	Cluster_53	Pseudomonas aeruginosa PAO1-VE13	66.6	Chromosome with gaps	Marshall University School of Medicine
GCA_000505965	Cluster_53	Pseudomonas aeruginosa JD314	66.7	Contig	University of Ottawa
GCA_000148745	Cluster_53	Pseudomonas aeruginosa 39016	65.5	Chromosome	Centre for Genomics Research, University of Liverpool
GCA_000481565	Cluster_53	Pseudomonas aeruginosa BWHPSA007	66.2	Scaffold	Broad Institute
GCA_000792895	Cluster_53	Pseudomonas aeruginosa 186	66.3	Contig	AstraZeneca
GCA_000793345	Cluster_53	Pseudomonas aeruginosa 208	66.2	Contig	AstraZeneca
GCA_000496325	Cluster_53	Pseudomonas aeruginosa VRFPA05	65.8	Contig	SANKARA NETHRALAYA, VISION RESEARCH FOUNDATION
GCA_000793765	Cluster_53	Pseudomonas aeruginosa 226	66.4	Contig	AstraZeneca
GCA_000789745	Cluster_53	Pseudomonas aeruginosa 26	65.9	Contig	AstraZeneca
GCA_000794095	Cluster_53	Pseudomonas aeruginosa 243	66.2	Contig	AstraZeneca
GCA_000412395	Cluster_53	Pseudomonas aeruginosa str. E2UoS	66.2	Contig	University of Strathclyde
GCA_000504045	Cluster_53	Pseudomonas aeruginosa MTB-1	66.2	Complete Genome	Tohoku University
GCA_000629245	Cluster_53	Pseudomonas aeruginosa 3577	66.4	Scaffold	Broad Institute
GCA_000794325	Cluster_53	Pseudomonas aeruginosa 252	66.2	Contig	AstraZeneca
GCA_000408865	Cluster_53	Pseudomonas aeruginosa PAK	66.4	Scaffold	University of Texas at Austin
GCA_000412375	Cluster_53	Pseudomonas aeruginosa str. MSH 3	66.4	Contig	University of Strathclyde
GCA_000481025	Cluster_53	Pseudomonas aeruginosa BL06	65.9	Scaffold	Broad Institute
GCA_000790965	Cluster_53	Pseudomonas aeruginosa 87	66.4	Contig	AstraZeneca
GCA_000796175	Cluster_53	Pseudomonas aeruginosa 353	66.5	Contig	AstraZeneca
GCA_000796165	Cluster_53	Pseudomonas aeruginosa 352	66.1	Contig	AstraZeneca

GCA_000629425	Cluster_53	Pseudomonas aeruginosa BWH058	66.1	Scaffold	Broad Institute
GCA_000792805	Cluster_53	Pseudomonas aeruginosa 181	65.8	Contig	AstraZeneca
GCA_000297295	Cluster_53	Pseudomonas aeruginosa ATCC 25324	65.9	Contig	University of Washington
GCA_000506205	Cluster_53	Pseudomonas aeruginosa JD324	66.5	Contig	University of Ottawa
GCA_000359565	Cluster_53	Pseudomonas aeruginosa PA45	66.3	Contig	CIBIO (Unitn)
GCA_000480395	Cluster_53	Pseudomonas aeruginosa C52	65.9	Scaffold	Broad Institute
GCA_000795625	Cluster_53	Pseudomonas aeruginosa 316	65.9	Contig	AstraZeneca
GCA_000455545	Cluster_53	Pseudomonas aeruginosa B3-1811	66.2	Contig	DTU
GCA_000795225	Cluster_53	Pseudomonas aeruginosa 296	66.4	Contig	AstraZeneca
GCA_000480945	Cluster_53	Pseudomonas aeruginosa BL10	66.3	Scaffold	Broad Institute
GCA_000341565	Cluster_53	Pseudomonas aeruginosa 18A	66.5	Contig	UNSW
GCA_000792305	Cluster_53	Pseudomonas aeruginosa 156	65.8	Contig	AstraZeneca
GCA_000797265	Cluster_53	Pseudomonas aeruginosa 407	66.5	Contig	AstraZeneca
GCA_000265035	Cluster_53	Pseudomonas aeruginosa XMG	66.4	Contig	Shanghai Jiao Tong University
GCA_000583955	Cluster_53	Pseudomonas aeruginosa LESB65	66.4	Chromosome with gaps	IBIS, University Laval
GCA_000794285	Cluster_53	Pseudomonas aeruginosa 250	66.2	Contig	AstraZeneca
GCA_000793725	Cluster_53	Pseudomonas aeruginosa 224	66.4	Contig	AstraZeneca
GCA_000794805	Cluster_53	Pseudomonas aeruginosa 275	66.0	Contig	AstraZeneca
GCA_000505985	Cluster_53	Pseudomonas aeruginosa JD315	66.5	Contig	University of Ottawa
GCA_000794005	Cluster_53	Pseudomonas aeruginosa 238	66.5	Contig	AstraZeneca
GCA_000794725	Cluster_53	Pseudomonas aeruginosa 271	65.3	Contig	AstraZeneca
GCA_000480355	Cluster_53	Pseudomonas aeruginosa CF614	66.0	Scaffold	Broad Institute
GCA_000790785	Cluster_53	Pseudomonas aeruginosa 78	66.2	Contig	AstraZeneca
GCA_000714515	Cluster_53	Pseudomonas aeruginosa PAO1H2O	66.3	Chromosome with gaps	University of Virginia
GCA_000629065	Cluster_53	Pseudomonas aeruginosa BWH033	66.0	Scaffold	Broad Institute
GCA_000481485	Cluster_53	Pseudomonas aeruginosa BWHPSA011	65.8	Scaffold	Broad Institute
GCA_000793705	Cluster_53	Pseudomonas aeruginosa 223	66.4	Contig	AstraZeneca
GCA_000481605	Cluster_53	Pseudomonas aeruginosa BWHPSA005	66.1	Scaffold	Broad Institute
GCA_000290555	Cluster_53	Pseudomonas aeruginosa PABL056	65.5	Scaffold	Northwestern University Feinberg School of Medicine
GCA_000481965	Cluster_53	Pseudomonas aeruginosa MSH10 1	66.4	Scaffold	Broad Institute
GCA_000796845	Cluster_53	Pseudomonas aeruginosa 386	65.8	Contig	AstraZeneca
GCA_000790725	Cluster_53	Pseudomonas aeruginosa 75	66.0	Contig	AstraZeneca
GCA_000520255	Cluster_53	Pseudomonas aeruginosa BWHPSA047	66.4	Scaffold	Broad Institute
GCA_000629565	Cluster_53	Pseudomonas aeruginosa BWH051	66.2	Scaffold	Broad Institute
GCA_000629165	Cluster_53	Pseudomonas aeruginosa 3581	66.0	Scaffold	Broad Institute
GCA_000796585	Cluster_53	Pseudomonas aeruginosa 373	66.5	Contig	AstraZeneca
GCA_000794265	Cluster_53	Pseudomonas aeruginosa 249	66.0	Contig	AstraZeneca
GCA_000796125	Cluster_53	Pseudomonas aeruginosa 350	66.1	Contig	AstraZeneca
GCA_000792325	Cluster_53	Pseudomonas aeruginosa 157	66.1	Contig	AstraZeneca
GCA_000223945	Cluster_53	Pseudomonas aeruginosa 19BR	66.1	Complete Genome	IBIS, Universite Laval
GCA_000481385	Cluster_53	Pseudomonas aeruginosa BWHPSA016	66.4	Scaffold	Broad Institute
GCA_000793315	Cluster_53	Pseudomonas aeruginosa 207	66.0	Contig	AstraZeneca
GCA_000793165	Cluster_53	Pseudomonas aeruginosa 199	66.5	Contig	AstraZeneca
GCA_000647615	Cluster_53	Pseudomonas aeruginosa ID4365	66.1	Contig	Universidad Nacional Autonoma de Mexico
GCA_000792675	Cluster_53	Pseudomonas aeruginosa 175	66.3	Contig	AstraZeneca
GCA_000412415	Cluster_53	Pseudomonas aeruginosa str. PA 62	66.3	Contig	University of Strathclyde
GCA_000481945	Cluster_53	Pseudomonas aeruginosa CF127	65.9	Scaffold	Broad Institute
GCA_000407905	Cluster_53	Pseudomonas aeruginosa MSH-10	66.4	Scaffold	Broad Institute
GCA_000792285	Cluster_53	Pseudomonas aeruginosa 155	66.3	Contig	AstraZeneca

GCA_000795435	Cluster_53	Pseudomonas aeruginosa 307	65.8	Contig	AstraZeneca
GCA_000789495	Cluster_53	Pseudomonas aeruginosa 14	66.1	Contig	AstraZeneca
GCA_000647635	Cluster_53	Pseudomonas aeruginosa IGB83	66.4	Contig	Universidad Nacional Autonoma de Mexico
GCA_000520415	Cluster_53	Pseudomonas aeruginosa BWHPSA039	66.0	Scaffold	Broad Institute
GCA_000481205	Cluster_53	Pseudomonas aeruginosa BWHPSA025	66.5	Scaffold	Broad Institute
GCA_000629285	Cluster_53	Pseudomonas aeruginosa 3575	66.0	Scaffold	Broad Institute
GCA_000796945	Cluster_53	Pseudomonas aeruginosa 391	66.4	Contig	AstraZeneca
GCA_000795635	Cluster_53	Pseudomonas aeruginosa 317	65.9	Contig	AstraZeneca
GCA_000794195	Cluster_53	Pseudomonas aeruginosa 247	66.1	Contig	AstraZeneca
GCA_000467675	Cluster_53	Pseudomonas aeruginosa VRFPA03	65.2	Contig	SANKARA NETHRALAYA, VISION RESEARCH FOUNDATION
GCA_000629345	Cluster_53	Pseudomonas aeruginosa PAO1-GFP	66.5	Scaffold	Broad Institute
GCA_000790625	Cluster_53	Pseudomonas aeruginosa 70	66.3	Contig	AstraZeneca
GCA_000480765	Cluster_53	Pseudomonas aeruginosa BL19	66.4	Scaffold	Broad Institute
GCA_000480665	Cluster_53	Pseudomonas aeruginosa BL24	66.1	Scaffold	Broad Institute
GCA_000481465	Cluster_53	Pseudomonas aeruginosa BWHPSA012	66.3	Scaffold	Broad Institute
GCA_000796475	Cluster_53	Pseudomonas aeruginosa 368	65.9	Contig	AstraZeneca
GCA_000793945	Cluster_53	Pseudomonas aeruginosa 235	66.0	Contig	AstraZeneca
GCA_000796025	Cluster_53	Pseudomonas aeruginosa 345	65.8	Contig	AstraZeneca
GCA_000797045	Cluster_53	Pseudomonas aeruginosa 396	66.4	Contig	AstraZeneca
GCA_000791105	Cluster_53	Pseudomonas aeruginosa 94	66.1	Contig	AstraZeneca
GCA_000797395	Cluster_53	Pseudomonas aeruginosa 413	65.5	Contig	AstraZeneca
GCA_000796385	Cluster_53	Pseudomonas aeruginosa 363	66.1	Contig	AstraZeneca
GCA_000797305	Cluster_53	Pseudomonas aeruginosa 409	66.4	Contig	AstraZeneca
GCA_000793685	Cluster_53	Pseudomonas aeruginosa 222	66.2	Contig	AstraZeneca
GCA_000792825	Cluster_53	Pseudomonas aeruginosa 182	66.2	Contig	AstraZeneca
GCA_000790935	Cluster_53	Pseudomonas aeruginosa 86	66.0	Contig	AstraZeneca
GCA_000698765	Cluster_53	Pseudomonas aeruginosa	66.5	Contig	International Centre for Genetic Engineering and Biotechnology
GCA_000568855	Cluster_53	Pseudomonas aeruginosa PAK 1	66.4	Scaffold	University of Texas at Austin
GCA_000790805	Cluster_53	Pseudomonas aeruginosa 79	66.3	Contig	AstraZeneca
GCA_000795465	Cluster_53	Pseudomonas aeruginosa 308	66.0	Contig	AstraZeneca
GCA_000629525	Cluster_53	Pseudomonas aeruginosa BWH053	66.2	Scaffold	Broad Institute
GCA_000480965	Cluster_53	Pseudomonas aeruginosa BL09	66.0	Scaffold	Broad Institute
GCA_000220025	Cluster_53	Pseudomonas aeruginosa AES-1R	66.5	Contig	Bacterial Pathogens in CF group, Sydney Medical School, The University of Sydney Sydney Australia 2006
GCA_000480645	Cluster_53	Pseudomonas aeruginosa BL25	66.3	Scaffold	Broad Institute
GCA_000790985	Cluster_53	Pseudomonas aeruginosa 88	66.0	Contig	AstraZeneca
GCA_000505945	Cluster_53	Pseudomonas aeruginosa JD312	66.6	Contig	University of Ottawa
GCA_000795685	Cluster_53	Pseudomonas aeruginosa 319	65.9	Contig	AstraZeneca
GCA_000506045	Cluster_53	Pseudomonas aeruginosa JD326	66.5	Contig	University of Ottawa
GCA_000506165	Cluster_53	Pseudomonas aeruginosa JD320	66.5	Contig	University of Ottawa
GCA_000481885	Cluster_53	Pseudomonas aeruginosa CF5	66.6	Scaffold	Broad Institute
GCA_000796985	Cluster_53	Pseudomonas aeruginosa 393	66.1	Contig	AstraZeneca
GCA_000794995	Cluster_53	Pseudomonas aeruginosa 285	66.5	Contig	AstraZeneca
GCA_000590905	Cluster_53	Pseudomonas aeruginosa MW3a	66.3	Contig	University of Malaya
GCA_000455405	Cluster_53	Pseudomonas aeruginosa B3-208	66.2	Contig	DTU
GCA_000797185	Cluster_53	Pseudomonas aeruginosa 403	66.2	Contig	AstraZeneca
GCA_000685845	Cluster_53	Pseudomonas aeruginosa NCAIM B.001380	66.1	Contig	DOE Joint Genome Institute
GCA_000226155	Cluster_53	Pseudomonas aeruginosa M18	66.5	Complete Genome	School of Life Sciences and Biotechnology, Shanghai Jiao Tong University, PR China
GCA_000789555	Cluster_53	Pseudomonas aeruginosa 18	65.6	Contig	AstraZeneca
GCA_000790765	Cluster_53	Pseudomonas aeruginosa 77	66.0	Contig	AstraZeneca

GCA_000481065	Cluster_53	Pseudomonas aeruginosa BL04	65.9	Scaffold	Broad Institute
GCA_000496605	Cluster_53	Pseudomonas aeruginosa PA1	66.3	Complete Genome	Department of Microbiology, The Third Military Medical University
GCA_000480495	Cluster_53	Pseudomonas aeruginosa C23	66.2	Scaffold	Broad Institute
GCA_000791275	Cluster_53	Pseudomonas aeruginosa 103	66.0	Contig	AstraZeneca
GCA_000626655	Cluster_53	Pseudomonas aeruginosa PA96	65.7	Chromosome with gaps	Universite Laval
GCA_000510305	Cluster_53	Pseudomonas aeruginosa SCV20265	66.3	Complete Genome	Helmholtz Center for Infection Research
GCA_000506305	Cluster_53	Pseudomonas aeruginosa JD331	66.5	Contig	University of Ottawa
GCA_000796745	Cluster_53	Pseudomonas aeruginosa 381	66.4	Contig	AstraZeneca
GCA_000259025	Cluster_53	Pseudomonas aeruginosa PADK2_CF510	66.1	Contig	DTU Systems Biology
GCA_000481325	Cluster_53	Pseudomonas aeruginosa BWHPSA019	66.4	Scaffold	Broad Institute
GCA_000796655	Cluster_53	Pseudomonas aeruginosa 377	66.5	Contig	AstraZeneca
GCA_000283055	Cluster_53	Pseudomonas aeruginosa DQ8	66.0	Contig	Shanghai Jiao Tong University
GCA_000797285	Cluster_53	Pseudomonas aeruginosa 408	66.4	Contig	AstraZeneca
GCA_000633495	Cluster_53	Pseudomonas aeruginosa H1l	66.4	Contig	University of Malaya
GCA_000791625	Cluster_53	Pseudomonas aeruginosa 120	66.4	Contig	AstraZeneca
GCA_000791325	Cluster_53	Pseudomonas aeruginosa 105	66.6	Contig	AstraZeneca
GCA_000794175	Cluster_53	Pseudomonas aeruginosa 245	65.6	Contig	AstraZeneca
GCA_000793455	Cluster_53	Pseudomonas aeruginosa 214	66.0	Contig	AstraZeneca
GCA_000791385	Cluster_53	Pseudomonas aeruginosa 108	66.0	Contig	AstraZeneca
GCA_000789925	Cluster_53	Pseudomonas aeruginosa 35	66.2	Contig	AstraZeneca
GCA_000796085	Cluster_53	Pseudomonas aeruginosa 348	66.3	Contig	AstraZeneca
GCA_000506025	Cluster_53	Pseudomonas aeruginosa JD323	66.6	Contig	University of Ottawa
GCA_000795235	Cluster_53	Pseudomonas aeruginosa 297	66.1	Contig	AstraZeneca
GCA_000481985	Cluster_53	Pseudomonas aeruginosa MSH3	66.4	Scaffold	Broad Institute
GCA_000455705	Cluster_53	Pseudomonas aeruginosa WC55	66.1	Contig	University of Illinois at Chicago
GCA_000505825	Cluster_53	Pseudomonas aeruginosa PK6	66.9	Contig	Saurashtra University
GCA_000791365	Cluster_53	Pseudomonas aeruginosa 107	66.4	Contig	AstraZeneca
GCA_000481505	Cluster_53	Pseudomonas aeruginosa BWHPSA010	66.1	Scaffold	Broad Institute
GCA_000480785	Cluster_53	Pseudomonas aeruginosa BL18	66.4	Scaffold	Broad Institute
GCA_000794625	Cluster_53	Pseudomonas aeruginosa 266	66.5	Contig	AstraZeneca
GCA_000794735	Cluster_53	Pseudomonas aeruginosa 272	66.1	Contig	AstraZeneca
GCA_000793385	Cluster_53	Pseudomonas aeruginosa 210	66.4	Contig	AstraZeneca
GCA_000567865	Cluster_53	Pseudomonas aeruginosa VRFPA06	65.8	Contig	SANKARA NETHRALAYA, VISION RESEARCH FOUNDATION
GCA_000796005	Cluster_53	Pseudomonas aeruginosa 344	66.2	Contig	AstraZeneca
GCA_000790305	Cluster_53	Pseudomonas aeruginosa 54	66.0	Contig	AstraZeneca
GCA_000481865	Cluster_53	Pseudomonas aeruginosa X24509	66.3	Scaffold	Broad Institute
GCA_000790505	Cluster_53	Pseudomonas aeruginosa 64	65.9	Contig	AstraZeneca
GCA_000794505	Cluster_53	Pseudomonas aeruginosa 260	66.1	Contig	AstraZeneca
GCA_000790605	Cluster_53	Pseudomonas aeruginosa 69	66.4	Contig	AstraZeneca
GCA_000791485	Cluster_53	Pseudomonas aeruginosa 113	65.9	Contig	AstraZeneca
GCA_000795905	Cluster_53	Pseudomonas aeruginosa 330	66.0	Contig	AstraZeneca
GCA_000795405	Cluster_53	Pseudomonas aeruginosa 305	66.4	Contig	AstraZeneca
GCA_000296325	Cluster_53	Pseudomonas aeruginosa PAO579	66.5	Contig	Marshall University School of Medicine
GCA_000505925	Cluster_53	Pseudomonas aeruginosa JD310	66.5	Contig	University of Ottawa
GCA_000789785	Cluster_53	Pseudomonas aeruginosa 28	66.1	Contig	AstraZeneca
GCA_000789645	Cluster_53	Pseudomonas aeruginosa 22	66.3	Contig	AstraZeneca
GCA_000705155	Cluster_53	Pseudomonas aeruginosa C1913C	66.2	Contig	University of Texas at Austin
GCA_000790325	Cluster_53	Pseudomonas aeruginosa 55	65.6	Contig	AstraZeneca
GCA_000481005	Cluster_53	Pseudomonas aeruginosa BL07	66.3	Scaffold	Broad Institute

GCA_000481405	Cluster_53	Pseudomonas aeruginosa BWHPSA015	66.5	Scaffold	Broad Institute
GCA_000795605	Cluster_53	Pseudomonas aeruginosa 315	66.1	Contig	AstraZeneca
GCA_000504485	Cluster_53	Pseudomonas aeruginosa ATCC 15442	66.2	Contig	State Key Laboratory of Microbial Metabolism and School of Life Sciences and Biotechnology, Shanghai Jiao Tong University
GCA_000506065	Cluster_53	Pseudomonas aeruginosa JD328	66.5	Contig	University of Ottawa
GCA_000794455	Cluster_53	Pseudomonas aeruginosa 258	66.0	Contig	AstraZeneca
GCA_000793985	Cluster_53	Pseudomonas aeruginosa 237	65.9	Contig	AstraZeneca
GCA_000792855	Cluster_53	Pseudomonas aeruginosa 184	65.9	Contig	AstraZeneca
GCA_000796095	Cluster_53	Pseudomonas aeruginosa 349	66.1	Contig	AstraZeneca
GCA_000496645	Cluster_53	Pseudomonas aeruginosa PA1R	66.3	Complete Genome	Department of Microbiology, The Third Military Medical University
GCA_000480595	Cluster_53	Pseudomonas aeruginosa M8A.2	66.4	Scaffold	Broad Institute
GCA_000796295	Cluster_53	Pseudomonas aeruginosa 359	66.0	Contig	AstraZeneca
GCA_000794705	Cluster_53	Pseudomonas aeruginosa 270	65.5	Contig	AstraZeneca
GCA_000481525	Cluster_53	Pseudomonas aeruginosa BWHPSA009	66.4	Scaffold	Broad Institute
GCA_000796725	Cluster_53	Pseudomonas aeruginosa 380	65.8	Contig	AstraZeneca
GCA_000172395	Cluster_53	Pseudomonas aeruginosa PAb1	66.8	Contig	The University of Maryland Center For Bioinformatics & Computational Biology
GCA_000503175	Cluster_53	Pseudomonas aeruginosa DHS29	65.8	Contig	Hospital of Besancon - France
GCA_000796255	Cluster_53	Pseudomonas aeruginosa 357	66.0	Contig	AstraZeneca
GCA_000412435	Cluster_53	Pseudomonas aeruginosa str. MSH 10	66.4	Contig	University of Strathclyde
GCA_000796905	Cluster_53	Pseudomonas aeruginosa 389	66.5	Contig	AstraZeneca
GCA_000796555	Cluster_53	Pseudomonas aeruginosa 372	66.4	Contig	AstraZeneca
GCA_000506225	Cluster_53	Pseudomonas aeruginosa JD325	66.6	Contig	University of Ottawa
GCA_000791985	Cluster_53	Pseudomonas aeruginosa 138	66.2	Contig	AstraZeneca
GCA_000793845	Cluster_53	Pseudomonas aeruginosa 230	66.3	Contig	AstraZeneca
GCA_000793055	Cluster_53	Pseudomonas aeruginosa 194	65.8	Contig	AstraZeneca
GCA_000793245	Cluster_53	Pseudomonas aeruginosa 203	66.3	Contig	AstraZeneca
GCA_000481425	Cluster_53	Pseudomonas aeruginosa BWHPSA014	66.5	Scaffold	Broad Institute
GCA_000797055	Cluster_53	Pseudomonas aeruginosa 397	66.4	Contig	AstraZeneca
GCA_000795085	Cluster_53	Pseudomonas aeruginosa 289	66.2	Contig	AstraZeneca
GCA_000480865	Cluster_53	Pseudomonas aeruginosa BL14	65.9	Scaffold	Broad Institute
GCA_000506125	Cluster_53	Pseudomonas aeruginosa JD316	66.6	Contig	University of Ottawa
GCA_000794825	Cluster_53	Pseudomonas aeruginosa 276	66.5	Contig	AstraZeneca
GCA_000750905	Cluster_53	Pseudomonas aeruginosa 5	65.9	Chromosome with gaps	IGH
GCA_000611995	Cluster_53	Pseudomonas aeruginosa PA99	66.1	Scaffold	Northwestern University Feinberg School of Medicine
GCA_000705195	Cluster_53	Pseudomonas aeruginosa C0324C	66.0	Contig	University of Texas at Austin
GCA_000790915	Cluster_53	Pseudomonas aeruginosa 85	66.2	Contig	AstraZeneca
GCA_000792725	Cluster_53	Pseudomonas aeruginosa 177	66.1	Contig	AstraZeneca
GCA_000794985	Cluster_53	Pseudomonas aeruginosa 284	66.1	Contig	AstraZeneca
GCA_000481045	Cluster_53	Pseudomonas aeruginosa BL05	66.5	Scaffold	Broad Institute
GCA_000790405	Cluster_53	Pseudomonas aeruginosa 59	66.1	Contig	AstraZeneca
GCA_000796645	Cluster_53	Pseudomonas aeruginosa 376	66.1	Contig	AstraZeneca
GCA_000629505	Cluster_53	Pseudomonas aeruginosa BWH054	65.9	Scaffold	Broad Institute
GCA_000297355	Cluster_53	Pseudomonas aeruginosa E2 1	66.4	Contig	University of Washington
GCA_000632755	Cluster_53	Pseudomonas otitidis	66.4	Scaffold	Liaoning University
GCA_000790345	Cluster_53	Pseudomonas aeruginosa 56	66.1	Contig	AstraZeneca
GCA_000629265	Cluster_53	Pseudomonas aeruginosa 3576	66.2	Scaffold	Broad Institute
GCA_000751715	Cluster_53	Pseudomonas aeruginosa 6	65.8	Contig	UB
GCA_000795875	Cluster_53	Pseudomonas aeruginosa 329	66.4	Contig	AstraZeneca
GCA_000790445	Cluster_53	Pseudomonas aeruginosa 61	66.4	Contig	AstraZeneca
GCA_000796705	Cluster_53	Pseudomonas aeruginosa 379	66.4	Contig	AstraZeneca

GCA_000629385	Cluster_53	Pseudomonas aeruginosa BWH060	66.2	Scaffold	Broad Institute
GCA_000481745	Cluster_53	Pseudomonas aeruginosa 6077	66.0	Scaffold	Broad Institute
GCA_000792625	Cluster_53	Pseudomonas aeruginosa 172	66.2	Contig	AstraZeneca
GCA_000791825	Cluster_53	Pseudomonas aeruginosa 130	66.5	Contig	AstraZeneca
GCA_000791915	Cluster_53	Pseudomonas aeruginosa 135	66.0	Contig	AstraZeneca
GCA_000794745	Cluster_53	Pseudomonas aeruginosa 273	65.9	Contig	AstraZeneca
GCA_000342145	Cluster_53	Pseudomonas aeruginosa PA21_ST175	66.1	Contig	Hospital Universitario 12 de Octubre
GCA_000481145	Cluster_53	Pseudomonas aeruginosa BWHPSA028	65.3	Scaffold	Broad Institute
GCA_000793955	Cluster_53	Pseudomonas aeruginosa 236	66.1	Contig	AstraZeneca
GCA_000792475	Cluster_53	Pseudomonas aeruginosa 165	66.3	Contig	AstraZeneca
GCA_000795205	Cluster_53	Pseudomonas aeruginosa 295	66.2	Contig	AstraZeneca
GCA_000794515	Cluster_53	Pseudomonas aeruginosa 261	65.9	Contig	AstraZeneca
GCA_000792515	Cluster_53	Pseudomonas aeruginosa 167	66.0	Contig	AstraZeneca
GCA_000790205	Cluster_53	Pseudomonas aeruginosa 49	66.3	Contig	AstraZeneca
GCA_000791585	Cluster_53	Pseudomonas aeruginosa 118	66.4	Contig	AstraZeneca
GCA_000795945	Cluster_53	Pseudomonas aeruginosa 341	65.8	Contig	AstraZeneca
GCA_000791705	Cluster_53	Pseudomonas aeruginosa 124	66.1	Contig	AstraZeneca
GCA_000793415	Cluster_53	Pseudomonas aeruginosa 212	66.2	Contig	AstraZeneca
GCA_000520235	Cluster_53	Pseudomonas aeruginosa BWHPSA048	66.4	Scaffold	Broad Institute
GCA_000629405	Cluster_53	Pseudomonas aeruginosa BWH059	66.1	Scaffold	Broad Institute
GCA_000414275	Cluster_53	Pseudomonas aeruginosa PAO1 1	66.4	Contig	Boise State University
GCA_000794785	Cluster_53	Pseudomonas aeruginosa 274	65.8	Contig	AstraZeneca
GCA_000794185	Cluster_53	Pseudomonas aeruginosa 246	66.0	Contig	AstraZeneca
GCA_000792635	Cluster_53	Pseudomonas aeruginosa 173	66.3	Contig	AstraZeneca
GCA_000480905	Cluster_53	Pseudomonas aeruginosa BL12	65.6	Scaffold	Broad Institute
GCA_000506265	Cluster_53	Pseudomonas aeruginosa JD329	66.4	Contig	University of Ottawa
GCA_000513235	Cluster_53	Pseudomonas aeruginosa MH27	65.9	Contig	CeBiTec
GCA_000791565	Cluster_53	Pseudomonas aeruginosa 117	66.2	Contig	AstraZeneca
GCA_000797025	Cluster_53	Pseudomonas aeruginosa 395	66.0	Contig	AstraZeneca
GCA_000797105	Cluster_53	Pseudomonas aeruginosa 399	66.4	Contig	AstraZeneca
GCA_000791235	Cluster_53	Pseudomonas aeruginosa 101	66.4	Contig	AstraZeneca
GCA_000152525	Cluster_53	Pseudomonas aeruginosa C3719	66.5	Scaffold	Broad Institute
GCA_000797225	Cluster_53	Pseudomonas aeruginosa 405	66.3	Contig	AstraZeneca
GCA_000629545	Cluster_53	Pseudomonas aeruginosa BWH052	66.2	Scaffold	Broad Institute
GCA_000480515	Cluster_53	Pseudomonas aeruginosa C20	66.2	Scaffold	Broad Institute
GCA_000790525	Cluster_53	Pseudomonas aeruginosa 65	66.5	Contig	AstraZeneca
GCA_000794365	Cluster_53	Pseudomonas aeruginosa 254	66.2	Contig	AstraZeneca
GCA_000793025	Cluster_53	Pseudomonas aeruginosa 192	66.1	Contig	AstraZeneca
GCA_000793645	Cluster_53	Pseudomonas aeruginosa 220	66.1	Contig	AstraZeneca
GCA_000793125	Cluster_53	Pseudomonas aeruginosa 197	66.4	Contig	AstraZeneca
GCA_000793295	Cluster_53	Pseudomonas aeruginosa 206	66.0	Contig	AstraZeneca
GCA_000793485	Cluster_53	Pseudomonas aeruginosa 215	65.8	Contig	AstraZeneca
GCA_000481545	Cluster_53	Pseudomonas aeruginosa BWHPSA008	66.3	Scaffold	Broad Institute
GCA_000480885	Cluster_53	Pseudomonas aeruginosa BL13	65.9	Scaffold	Broad Institute
GCA_000791125	Cluster_53	Pseudomonas aeruginosa 95	66.1	Contig	AstraZeneca
GCA_000414255	Cluster_53	Pseudomonas aeruginosa PAO1-CipR	66.1	Contig	Boise State University
GCA_000795815	Cluster_53	Pseudomonas aeruginosa 326	66.1	Contig	AstraZeneca
GCA_000790115	Cluster_53	Pseudomonas aeruginosa 45	66.5	Contig	AstraZeneca
GCA_000481645	Cluster_53	Pseudomonas aeruginosa BWHPSA003	66.0	Scaffold	Broad Institute

GCA_000412295	Cluster_53	Pseudomonas aeruginosa str. C 1334	66.4	Contig	University of Strathclyde
GCA_000791945	Cluster_53	Pseudomonas aeruginosa 136	66.0	Contig	AstraZeneca
GCA_000789535	Cluster_53	Pseudomonas aeruginosa 16	66.1	Contig	AstraZeneca
GCA_000792385	Cluster_53	Pseudomonas aeruginosa 160	66.1	Contig	AstraZeneca
GCA_000795665	Cluster_53	Pseudomonas aeruginosa 318	66.1	Contig	AstraZeneca
GCA_000481805	Cluster_53	Pseudomonas aeruginosa JJ692	66.1	Scaffold	Broad Institute
GCA_000790145	Cluster_53	Pseudomonas aeruginosa 46	65.6	Contig	AstraZeneca
GCA_000792225	Cluster_53	Pseudomonas aeruginosa 150	66.2	Contig	AstraZeneca
GCA_000629585	Cluster_53	Pseudomonas aeruginosa BWH050	66.3	Scaffold	Broad Institute
GCA_000792165	Cluster_53	Pseudomonas aeruginosa 148	65.8	Contig	AstraZeneca
GCA_000792345	Cluster_53	Pseudomonas aeruginosa 158	66.1	Contig	AstraZeneca
GCA_000629305	Cluster_53	Pseudomonas aeruginosa 3574	66.4	Scaffold	Broad Institute
GCA_000568235	Cluster_53	Pseudomonas aeruginosa CF_PA39	66.4	Contig	Vrije Universiteit Brussel
GCA_000796825	Cluster_53	Pseudomonas aeruginosa 385	66.3	Contig	AstraZeneca
GCA_000792095	Cluster_53	Pseudomonas aeruginosa 144	66.5	Contig	AstraZeneca
GCA_000796405	Cluster_53	Pseudomonas aeruginosa 364	66.0	Contig	AstraZeneca
GCA_000795485	Cluster_53	Pseudomonas aeruginosa 309	65.7	Contig	AstraZeneca
GCA_000404265	Cluster_53	Pseudomonas aeruginosa PA14	66.3	Scaffold	Broad Institute
GCA_000791805	Cluster_53	Pseudomonas aeruginosa 129	65.8	Contig	AstraZeneca
GCA_000796225	Cluster_53	Pseudomonas aeruginosa 355	66.1	Contig	AstraZeneca
GCA_000796285	Cluster_53	Pseudomonas aeruginosa 358	65.9	Contig	AstraZeneca
GCA_000795955	Cluster_53	Pseudomonas aeruginosa 342	66.0	Contig	AstraZeneca
GCA_000480825	Cluster_53	Pseudomonas aeruginosa BL16	66.0	Scaffold	Broad Institute
GCA_000756575	Cluster_53	Pseudomonas aeruginosa 7	66.3	Contig	UDRI
GCA_000789885	Cluster_53	Pseudomonas aeruginosa 33	65.9	Contig	AstraZeneca
GCA_000793595	Cluster_53	Pseudomonas aeruginosa 218	66.4	Contig	AstraZeneca
GCA_000793805	Cluster_53	Pseudomonas aeruginosa 228	66.1	Contig	AstraZeneca
GCA_000482025	Cluster_53	Pseudomonas aeruginosa 62	66.3	Scaffold	Broad Institute
GCA_000795385	Cluster_53	Pseudomonas aeruginosa 304	66.1	Contig	AstraZeneca
GCA_000790655	Cluster_53	Pseudomonas aeruginosa 72	66.4	Contig	AstraZeneca
GCA_000792665	Cluster_53	Pseudomonas aeruginosa 174	66.0	Contig	AstraZeneca
GCA_000792605	Cluster_53	Pseudomonas aeruginosa 171	66.0	Contig	AstraZeneca
GCA_000789705	Cluster_53	Pseudomonas aeruginosa 24	66.0	Contig	AstraZeneca
GCA_000789755	Cluster_53	Pseudomonas aeruginosa 27	66.4	Contig	AstraZeneca
GCA_000568115	Cluster_53	Pseudomonas aeruginosa RB-48	66.5	Contig	UNIVERSITY OF MALAYA
GCA_000793225	Cluster_53	Pseudomonas aeruginosa 202	66.2	Contig	AstraZeneca
GCA_000439855	Cluster_53	Pseudomonas aeruginosa LCT-PA220	66.2	Scaffold	BGI Research Institute
GCA_000481365	Cluster_53	Pseudomonas aeruginosa BWHPSA017	66.0	Scaffold	Broad Institute
GCA_000791065	Cluster_53	Pseudomonas aeruginosa 92	66.2	Contig	AstraZeneca
GCA_000481825	Cluster_53	Pseudomonas aeruginosa S54485	66.0	Scaffold	Broad Institute
GCA_000481905	Cluster_53	Pseudomonas aeruginosa CF27	66.3	Scaffold	Broad Institute
GCA_000480985	Cluster_53	Pseudomonas aeruginosa BL08	65.9	Scaffold	Broad Institute
GCA_000794845	Cluster_53	Pseudomonas aeruginosa 277	66.1	Contig	AstraZeneca
GCA_000797245	Cluster_53	Pseudomonas aeruginosa 406	66.2	Contig	AstraZeneca
GCA_000506005	Cluster_53	Pseudomonas aeruginosa JD318	66.5	Contig	University of Ottawa
GCA_000481685	Cluster_53	Pseudomonas aeruginosa BWHPSA001	66.4	Scaffold	Broad Institute
GCA_000247455	Cluster_53	Pseudomonas aeruginosa MPAO1/P2	66.5	Contig	Argonne National Laboratory
GCA_000790025	Cluster_53	Pseudomonas aeruginosa 40	66.0	Contig	AstraZeneca
GCA_000791085	Cluster_53	Pseudomonas aeruginosa 93	65.8	Contig	AstraZeneca

GCA_000795505	Cluster_53	Pseudomonas aeruginosa 310	66.0	Contig	AstraZeneca
GCA_000795525	Cluster_53	Pseudomonas aeruginosa 311	65.9	Contig	AstraZeneca
GCA_000790085	Cluster_53	Pseudomonas aeruginosa 43	66.2	Contig	AstraZeneca
GCA_000790155	Cluster_53	Pseudomonas aeruginosa 47	66.6	Contig	AstraZeneca
GCA_000615485	Cluster_53	Pseudomonas aeruginosa JCM 5962	66.3	Contig	The University of Tokyo
GCA_000017205	Cluster_53	Pseudomonas aeruginosa PA7	66.4	Complete Genome	J. Craig Venter Institute
GCA_000796955	Cluster_53	Pseudomonas aeruginosa 392	66.1	Contig	AstraZeneca
GCA_000520455	Cluster_53	Pseudomonas aeruginosa BWHPSA037	65.6	Scaffold	Broad Institute
GCA_000287815	Cluster_53	Pseudomonas aeruginosa N002	66.8	Contig	CSIR-NEIST, Jorhat
GCA_000795845	Cluster_53	Pseudomonas aeruginosa 327	66.1	Contig	AstraZeneca
GCA_000480415	Cluster_53	Pseudomonas aeruginosa C51	66.1	Scaffold	Broad Institute
GCA_000797165	Cluster_53	Pseudomonas aeruginosa 402	66.3	Contig	AstraZeneca
GCA_000297275	Cluster_53	Pseudomonas aeruginosa ATCC 14886	66.4	Contig	University of Washington
GCA_000468935	Cluster_53	Pseudomonas aeruginosa c7447m	66.5	Chromosome with gaps	Marshall University School of Medicine
GCA_000789965	Cluster_53	Pseudomonas aeruginosa 37	66.5	Contig	AstraZeneca
GCA_000412535	Cluster_53	Pseudomonas aeruginosa str. C 1433	66.3	Contig	University of Strathclyde
GCA_000794925	Cluster_53	Pseudomonas aeruginosa 281	65.8	Contig	AstraZeneca
GCA_000282915	Cluster_53	Pseudomonas aeruginosa MRW44.1	66.5	Contig	Michigan State University
GCA_000297335	Cluster_53	Pseudomonas aeruginosa CI27	66.1	Contig	University of Washington
GCA_000795925	Cluster_53	Pseudomonas aeruginosa 340	66.5	Contig	AstraZeneca
GCA_000520375	Cluster_53	Pseudomonas aeruginosa BWHPSA041	65.9	Scaffold	Broad Institute
GCA_000789995	Cluster_53	Pseudomonas aeruginosa 39	66.1	Contig	AstraZeneca
GCA_000506885	Cluster_53	Pseudomonas aeruginosa VRFPA08	66.1	Contig	SANKARA NETHRALAYA, VISION RESEARCH FOUNDATION
GCA_000794045	Cluster_53	Pseudomonas aeruginosa 240	66.3	Contig	AstraZeneca
GCA_000480555	Cluster_53	Pseudomonas aeruginosa M8A.4	66.5	Scaffold	Broad Institute
GCA_000791835	Cluster_53	Pseudomonas aeruginosa 131	65.9	Contig	AstraZeneca
GCA_000797345	Cluster_53	Pseudomonas aeruginosa 411	65.8	Contig	AstraZeneca
GCA_000796325	Cluster_53	Pseudomonas aeruginosa 360	66.6	Contig	AstraZeneca
GCA_000481185	Cluster_53	Pseudomonas aeruginosa BWHPSA026	66.0	Scaffold	Broad Institute
GCA_000359505	Cluster_53	Pseudomonas aeruginosa B136-33	66.4	Complete Genome	National Tsing Hua University
GCA_000506365	Cluster_53	Pseudomonas aeruginosa JD335	66.4	Contig	University of Ottawa
GCA_000481085	Cluster_53	Pseudomonas aeruginosa BL03	66.1	Scaffold	Broad Institute
GCA_000026645	Cluster_53	Pseudomonas aeruginosa LESB58	66.3	Complete Genome	Wellcome Trust Sanger Institute
GCA_000791465	Cluster_53	Pseudomonas aeruginosa 112	66.4	Contig	AstraZeneca
GCA_000793085	Cluster_53	Pseudomonas aeruginosa 195	66.1	Contig	AstraZeneca
GCA_000629225	Cluster_53	Pseudomonas aeruginosa 3578	66.2	Scaffold	Broad Institute
GCA_000710625	Cluster_53	Pseudomonas aeruginosa 2	66.5	Contig	Walter Reed Army Institute of Research
GCA_000792245	Cluster_53	Pseudomonas aeruginosa 153	66.5	Contig	AstraZeneca
GCA_000793005	Cluster_53	Pseudomonas aeruginosa 191	66.1	Contig	AstraZeneca
GCA_000789625	Cluster_53	Pseudomonas aeruginosa 20	65.9	Contig	AstraZeneca
GCA_000795265	Cluster_53	Pseudomonas aeruginosa 298	66.1	Contig	AstraZeneca
GCA_000792205	Cluster_53	Pseudomonas aeruginosa 149	66.0	Contig	AstraZeneca
GCA_000796525	Cluster_53	Pseudomonas aeruginosa 370	66.2	Contig	AstraZeneca
GCA_000790185	Cluster_53	Pseudomonas aeruginosa 48	65.8	Contig	AstraZeneca
GCA_000792025	Cluster_53	Pseudomonas aeruginosa 140	66.4	Contig	AstraZeneca
GCA_000689435	Cluster_53	Pseudomonas aeruginosa MH38	65.8	Contig	CEBITEC
GCA_000558345	Cluster_53	Pseudomonas aeruginosa VRFPA09	65.3	Contig	SANKARA NETHRALAYA, VISION RESEARCH FOUNDATION
GCA_000795805	Cluster_53	Pseudomonas aeruginosa 325	66.0	Contig	AstraZeneca
GCA_000792785	Cluster_53	Pseudomonas aeruginosa 180	66.0	Contig	AstraZeneca

GCA_000797205	Cluster_53	Pseudomonas aeruginosa 404	66.0	Contig	AstraZeneca
GCA_000481765	Cluster_53	Pseudomonas aeruginosa 19660	66.2	Scaffold	Broad Institute
GCA_000791345	Cluster_53	Pseudomonas aeruginosa 106	66.5	Contig	AstraZeneca
GCA_000520315	Cluster_53	Pseudomonas aeruginosa BWHPSA044	66.0	Scaffold	Broad Institute
GCA_000797145	Cluster_53	Pseudomonas aeruginosa 401	66.3	Contig	AstraZeneca
GCA_000223925	Cluster_53	Pseudomonas aeruginosa 9BR	66.1	Contig	IBIS, Universite Laval
GCA_000520295	Cluster_53	Pseudomonas aeruginosa BWHPSA045	65.8	Scaffold	Broad Institute
GCA_000790565	Cluster_53	Pseudomonas aeruginosa 67	66.1	Contig	AstraZeneca
GCA_000795425	Cluster_53	Pseudomonas aeruginosa 306	66.1	Contig	AstraZeneca
GCA_000795305	Cluster_53	Pseudomonas aeruginosa 300	65.9	Contig	AstraZeneca
GCA_000793825	Cluster_53	Pseudomonas aeruginosa 229	66.0	Contig	AstraZeneca
GCA_000791965	Cluster_53	Pseudomonas aeruginosa 137	66.1	Contig	AstraZeneca
GCA_000790355	Cluster_53	Pseudomonas aeruginosa 57	65.5	Contig	AstraZeneca
GCA_000481705	Cluster_53	Pseudomonas aeruginosa X13273	66.0	Scaffold	Broad Institute
GCA_000794885	Cluster_53	Pseudomonas aeruginosa 279	65.6	Contig	AstraZeneca
GCA_000796795	Cluster_53	Pseudomonas aeruginosa 384	66.3	Contig	AstraZeneca
GCA_000482005	Cluster_53	Pseudomonas aeruginosa E2	66.4	Scaffold	Broad Institute
GCA_000520335	Cluster_53	Pseudomonas aeruginosa BWHPSA043	66.0	Scaffold	Broad Institute
GCA_000794865	Cluster_53	Pseudomonas aeruginosa 278	65.8	Contig	AstraZeneca
GCA_000480615	Cluster_53	Pseudomonas aeruginosa M8A.1	66.4	Scaffold	Broad Institute
GCA_000737795	Cluster_53	Pseudomonas aeruginosa 3	66.0	Contig	Hospital Universitario 12 de Octubre
GCA_000792115	Cluster_53	Pseudomonas aeruginosa 145	66.1	Contig	AstraZeneca
GCA_000629445	Cluster_53	Pseudomonas aeruginosa BWH057	66.0	Scaffold	Broad Institute
GCA_000794165	Cluster_53	Pseudomonas aeruginosa 244	66.0	Contig	AstraZeneca
GCA_000481285	Cluster_53	Pseudomonas aeruginosa BWHPSA021	66.3	Scaffold	Broad Institute
GCA_000505805	Cluster_53	Pseudomonas aeruginosa PFK10	66.8	Contig	Saurashtra University
GCA_000795365	Cluster_53	Pseudomonas aeruginosa 303	65.6	Contig	AstraZeneca
GCA_000506105	Cluster_53	Pseudomonas aeruginosa JD313	66.5	Contig	University of Ottawa
GCA_000629605	Cluster_53	Pseudomonas aeruginosa BWH049	66.3	Scaffold	Broad Institute
GCA_000271985	Cluster_53	Pseudomonas aeruginosa SJTD-1	66.8	Contig	Shanghai Jiao Tong University
GCA_000797355	Cluster_53	Pseudomonas aeruginosa 412	66.2	Contig	AstraZeneca
GCA_000795765	Cluster_53	Pseudomonas aeruginosa 323	66.0	Contig	AstraZeneca
GCA_000794945	Cluster_53	Pseudomonas aeruginosa 282	66.0	Contig	AstraZeneca
GCA_000791785	Cluster_53	Pseudomonas aeruginosa 128	66.4	Contig	AstraZeneca
GCA_000760495	Cluster_53	Pseudomonas aeruginosa 11	66.0	Contig	Center for Cellular and Molecular Biology(CCMB)
GCA_000790385	Cluster_53	Pseudomonas aeruginosa 58	66.5	Contig	AstraZeneca
GCA_000789935	Cluster_53	Pseudomonas aeruginosa 36	65.8	Contig	AstraZeneca
GCA_000794055	Cluster_53	Pseudomonas aeruginosa 241	66.1	Contig	AstraZeneca
GCA_000705215	Cluster_53	Pseudomonas aeruginosa C2159M	66.5	Contig	University of Texas at Austin
GCA_000793045	Cluster_53	Pseudomonas aeruginosa 193	66.1	Contig	AstraZeneca
GCA_000480805	Cluster_53	Pseudomonas aeruginosa BL17	66.0	Scaffold	Broad Institute
GCA_000006765	Cluster_53	Pseudomonas aeruginosa PAO1	66.6	Complete Genome	PathoGenesis Corporation
GCA_000793915	Cluster_53	Pseudomonas aeruginosa 234	66.2	Contig	AstraZeneca
GCA_000412735	Cluster_53	Pseudomonas aeruginosa PGPR2	66.0	Contig	Madurai Kamaraj University
GCA_000412555	Cluster_53	Pseudomonas aeruginosa str. C 1426	66.4	Contig	University of Strathclyde
GCA_000789905	Cluster_53	Pseudomonas aeruginosa 34	66.1	Contig	AstraZeneca
GCA_000481785	Cluster_53	Pseudomonas aeruginosa U2504	65.9	Scaffold	Broad Institute
GCA_000794335	Cluster_53	Pseudomonas aeruginosa 253	65.4	Contig	AstraZeneca
GCA_000796765	Cluster_53	Pseudomonas aeruginosa 382	66.5	Contig	AstraZeneca

GCA_000792765	Cluster_53	Pseudomonas aeruginosa 179	66.1	Contig	AstraZeneca
GCA_000791735	Cluster_53	Pseudomonas aeruginosa 126	66.2	Contig	AstraZeneca
GCA_000797325	Cluster_53	Pseudomonas aeruginosa 410	66.5	Contig	AstraZeneca
GCA_000520215	Cluster_53	Pseudomonas aeruginosa Z61	66.3	Scaffold	Broad Institute
GCA_000480925	Cluster_53	Pseudomonas aeruginosa BL11	65.9	Scaffold	Broad Institute
GCA_000791205	Cluster_53	Pseudomonas aeruginosa 99	66.1	Contig	AstraZeneca
GCA_000629485	Cluster_53	Pseudomonas aeruginosa BWH055	66.3	Scaffold	Broad Institute
GCA_000790865	Cluster_53	Pseudomonas aeruginosa 82	66.4	Contig	AstraZeneca
GCA_000792975	Cluster_53	Pseudomonas aeruginosa 190	66.1	Contig	AstraZeneca
GCA_000572265	Cluster_53	Pseudomonas aeruginosa BK1	66.3	Contig	Aravind Medical Research Foundation
GCA_000615565	Cluster_53	Pseudomonas aeruginosa JCM 14847	66.3	Contig	The University of Tokyo
GCA_000794545	Cluster_53	Pseudomonas aeruginosa 262	65.6	Contig	AstraZeneca
GCA_000583915	Cluster_53	Pseudomonas aeruginosa LESlike7	66.5	Chromosome with gaps	IBIS, University Laval
GCA_000506805	Cluster_53	Pseudomonas aeruginosa VRFPA07	65.9	Contig	SANKARA NETHRALAYA, VISION RESEARCH FOUNDATION
GCA_000506085	Cluster_53	Pseudomonas aeruginosa JD306	66.4	Contig	University of Ottawa
GCA_000629105	Cluster_53	Pseudomonas aeruginosa BWH031	66.2	Scaffold	Broad Institute
GCA_000792575	Cluster_53	Pseudomonas aeruginosa 170	66.4	Contig	AstraZeneca
GCA_000611975	Cluster_53	Pseudomonas aeruginosa PA103	66.1	Scaffold	Northwestern University Feinberg School of Medicine
GCA_000795785	Cluster_53	Pseudomonas aeruginosa 324	66.3	Contig	AstraZeneca
GCA_000790065	Cluster_53	Pseudomonas aeruginosa 42	66.2	Contig	AstraZeneca
GCA_000792925	Cluster_53	Pseudomonas aeruginosa 187	66.4	Contig	AstraZeneca
GCA_000786485	Cluster_53	Pseudomonas aeruginosa WS394	66.1	Contig	CEBITEC
GCA_000789845	Cluster_53	Pseudomonas aeruginosa 32	65.9	Contig	AstraZeneca
GCA_000793285	Cluster_53	Pseudomonas aeruginosa 205	66.3	Contig	AstraZeneca
GCA_000793505	Cluster_53	Pseudomonas aeruginosa 216	65.7	Contig	AstraZeneca
GCA_000412515	Cluster_53	Pseudomonas aeruginosa str. J 1385	66.1	Contig	University of Strathclyde
GCA_000796605	Cluster_53	Pseudomonas aeruginosa 374	65.9	Contig	AstraZeneca
GCA_000791005	Cluster_53	Pseudomonas aeruginosa 89	66.0	Contig	AstraZeneca
GCA_000790425	Cluster_53	Pseudomonas aeruginosa 60	65.9	Contig	AstraZeneca
GCA_000794675	Cluster_53	Pseudomonas aeruginosa 269	66.2	Contig	AstraZeneca
GCA_000791905	Cluster_53	Pseudomonas aeruginosa 134	65.9	Contig	AstraZeneca
GCA_000796785	Cluster_53	Pseudomonas aeruginosa 383	66.4	Contig	AstraZeneca
GCA_000796445	Cluster_53	Pseudomonas aeruginosa 366	66.5	Contig	AstraZeneca
GCA_000505905	Cluster_53	Pseudomonas aeruginosa JD304	66.5	Contig	University of Ottawa
GCA_000793905	Cluster_53	Pseudomonas aeruginosa 233	66.4	Contig	AstraZeneca
GCA_000794425	Cluster_53	Pseudomonas aeruginosa 256	66.5	Contig	AstraZeneca
GCA_000480535	Cluster_53	Pseudomonas aeruginosa M9A.1	66.4	Scaffold	Broad Institute
GCA_000790705	Cluster_53	Pseudomonas aeruginosa 74	65.8	Contig	AstraZeneca
GCA_000481165	Cluster_53	Pseudomonas aeruginosa BWHPSA027	66.0	Scaffold	Broad Institute
GCA_000790905	Cluster_53	Pseudomonas aeruginosa 84	66.0	Contig	AstraZeneca
GCA_000792845	Cluster_53	Pseudomonas aeruginosa 183	65.9	Contig	AstraZeneca
GCA_000629085	Cluster_53	Pseudomonas aeruginosa BWH032	66.3	Scaffold	Broad Institute
GCA_000743405	Cluster_53	Pseudomonas aeruginosa 4	66.1	Scaffold	Los Alamos National Laboratory
GCA_000790285	Cluster_53	Pseudomonas aeruginosa 53	66.5	Contig	AstraZeneca
GCA_000796685	Cluster_53	Pseudomonas aeruginosa 378	66.4	Contig	AstraZeneca
GCA_000480705	Cluster_53	Pseudomonas aeruginosa BL22	66.1	Scaffold	Broad Institute
GCA_000792465	Cluster_53	Pseudomonas aeruginosa 164	66.1	Contig	AstraZeneca
GCA_000468555	Cluster_53	Pseudomonas aeruginosa PAO581	66.5	Chromosome with gaps	Marshall University School of Medicine
GCA_000790885	Cluster_53	Pseudomonas aeruginosa 83	66.1	Contig	AstraZeneca

GCA_000287875	Cluster_53	Pseudomonas aeruginosa AH16	66.1	Contig	Anhui University of Traditional Chinese Medicine
GCA_000790545	Cluster_53	Pseudomonas aeruginosa 66	66.1	Contig	AstraZeneca
GCA_000792445	Cluster_53	Pseudomonas aeruginosa 163	66.2	Contig	AstraZeneca
GCA_000794555	Cluster_53	Pseudomonas aeruginosa 263	66.1	Contig	AstraZeneca
GCA_000794905	Cluster_53	Pseudomonas aeruginosa 280	65.6	Contig	AstraZeneca
GCA_000508765	Cluster_53	Pseudomonas aeruginosa LES431	66.3	Complete Genome	IBIS, University Laval
GCA_000795325	Cluster_53	Pseudomonas aeruginosa 301	66.4	Contig	AstraZeneca
GCA_000455425	Cluster_53	Pseudomonas aeruginosa B3-CFI	66.2	Contig	DTU
GCA_000796205	Cluster_53	Pseudomonas aeruginosa 354	65.8	Contig	AstraZeneca
GCA_000506145	Cluster_53	Pseudomonas aeruginosa JD317	66.5	Contig	University of Ottawa
GCA_000790105	Cluster_53	Pseudomonas aeruginosa 44	65.7	Contig	AstraZeneca
GCA_000790265	Cluster_53	Pseudomonas aeruginosa 52	66.1	Contig	AstraZeneca
GCA_000510745	Cluster_54	Pseudomonas sp. MOIL14HWK12:I2	66.2	Scaffold	JGI
GCA_000510705	Cluster_54	Pseudomonas sp. MOIL14HWK12:I1	66.3	Scaffold	JGI
GCA_000236825	Cluster_54	Pseudomonas psychrotolerans L19	65.7	Contig	Soil, Water and Environmental Science, University of Arizona
GCA_000730625	Cluster_54	Pseudomonas oryzihabitans NBRC 102199	66.2	Contig	National Institute of Technology and Evaluation
GCA_000316965	Cluster_54	Pseudomonas sp. 313	65.3	Contig	San Diego State University
GCA_000510765	Cluster_54	Pseudomonas oleovorans MOIL14HWK12	66.3	Scaffold	JGI
GCA_000280765	Singleton	Pseudomonas gingeri NCPPB 3146	62.6	Scaffold	University of Oxford
GCA_000009225	Singleton	Pseudomonas fluorescens SBW25	60.5	Complete Genome	Wellcome Trust Sanger Institute
GCA_000166515	Singleton	Pseudomonas fluorescens WH6	60.6	Chromosome	Oregon State University
GCA_000281895	Singleton	Pseudomonas fluorescens Q2-87	60.6	Chromosome	USDA - Agricultural Research Service, USA
GCA_000293885	Singleton	Pseudomonas fluorescens NCIMB 11764	59.0	Chromosome	University of North Texas
GCA_000217955	Singleton	Pseudomonas fluorescens HK44	58.7	Scaffold	Center for Environmental Biotechnology
GCA_000280805	Singleton	Pseudomonas fluorescens NZ007	60.0	Scaffold	The Sainsbury Laboratory
GCA_000346775	Singleton	Pseudomonas fluorescens Pf29Arp	60.9	Scaffold	INRA
GCA_000308175	Singleton	Pseudomonas fluorescens BS2	60.6	Contig	LSHTM
GCA_000019125	Singleton	Pseudomonas putida GB-1	61.9	Complete Genome	US DOE Joint Genome Institute (JGI-PGF)
GCA_000019445	Singleton	Pseudomonas putida W619	61.4	Complete Genome	US DOE Joint Genome Institute
GCA_000319305	Singleton	Pseudomonas putida CSV86	63.1	Contig	National Environmental Engineering Research Institute (NEERI)
GCA_000452865	Singleton	Pseudomonas syringae UB246	57.1	Scaffold	University of Arizona
GCA_000026105	Singleton	Pseudomonas entomophila L48	64.2	Complete Genome	Genoscope
GCA_000661915	Singleton	Pseudomonas stutzeri	62.2	Complete Genome	Univesitat de les Illes Balears (UIB)
GCA_000307775	Singleton	Pseudomonas stutzeri KOS6	62.9	Scaffold	RIPCM
GCA_000455665	Singleton	Pseudomonas stutzeri MF28	62.3	Contig	University of Illinois at Chicago
GCA_000145845	Singleton	Pseudomonas syringae pv. maculicola str. ES4326	58.5	Scaffold	University of North Carolina at Chapel Hill
GCA_000585995	Singleton	Pseudomonas brassicacearum	60.5	Complete Genome	University of Manitoba
GCA_000262065	Singleton	Pseudomonas pseudoalcaligenes KF707	65.3	Contig	Dipartimento di Bioscienze, Biotecnologie e Scienze Farmacologiche
GCA_000626735	Singleton	Pseudomonas pseudoalcaligenes AD6	62.5	Contig	University of Minnesota
GCA_000280785	Singleton	Pseudomonas agarici NCPPB 2289	59.1	Scaffold	The Sainsbury Laboratory
GCA_000242115	Singleton	Pseudomonas extremaustralis 14-3 substr. 14-3b	60.7	Contig	INDEAR
GCA_000250615	Singleton	Pseudomonas fragi B25	59.4	Scaffold	Nanjing Normal University
GCA_000364705	Singleton	Pseudomonas fuscovaginae SE-1	63.1	Contig	Colorado State University
GCA_000467025	Singleton	Pseudomonas fuscovaginae DAR 77800	61.1	Contig	Charles Sturt University
GCA_000689415	Singleton	Pseudomonas knackmussii B13	65.6	Complete Genome	CHUV-UNIL
GCA_000508205	Singleton	Pseudomonas sp. TKP	60.5	Complete Genome	Tohoku University
GCA_000478505	Singleton	Pseudomonas sp. HPB0071	55.2	Scaffold	Broad Institute
GCA_000483465	Singleton	Pseudomonas sp. LAIL14HWK12:I7	62.5	Scaffold	DOE Joint Genome Institute
GCA_000514375	Singleton	Pseudomonas sp. LAMO17WK12:I2	61.0	Scaffold	DOE Joint Genome Institute

GCA_000514395	Singleton	Pseudomonas sp. URMO17WK12:I12	59.1	Scaffold	DOE Joint Genome Institute
GCA_000620245	Singleton	Pseudomonas sp. URHB0015	60.6	Scaffold	DOE Joint Genome Institute
GCA_000620365	Singleton	Pseudomonas sp. URMO17WK12:I8	62.8	Scaffold	DOE Joint Genome Institute
GCA_000222125	Singleton	Pseudomonas sp. S9	56.5	Contig	Key Laboratory of Marine Biogenetic Resources, Third Institute of Oceanography, State Oceanic Administration (SOA), PR China
GCA_000263855	Singleton	Pseudomonas sp. M47T1	62.5	Contig	University of Coimbra - Faculty of Life Sciences
GCA_000282215	Singleton	Pseudomonas sp. GM21	58.5	Contig	Oak Ridge National Lab
GCA_000282395	Singleton	Pseudomonas sp. GM55	59.7	Contig	Oak Ridge National Lab
GCA_000282515	Singleton	Pseudomonas sp. GM80	59.2	Contig	Oak Ridge National Lab
GCA_000282535	Singleton	Pseudomonas sp. GM84	63.2	Contig	Oak Ridge National Lab
GCA_000306015	Singleton	Pseudomonas sp. Chol1	64.0	Contig	University of Muenster
GCA_000346755	Singleton	Pseudomonas sp. CBZ-4	61.2	Contig	Harbin Institute of Technology
GCA_000416155	Singleton	Pseudomonas sp. CF149	57.5	Contig	Boise State University
GCA_000416215	Singleton	Pseudomonas sp. CF161	62.6	Contig	Boise State University
GCA_000418555	Singleton	Pseudomonas sp. P818	63.4	Contig	Institute of Crop Sciences, Chinese Academy of Agricultural Sciences
GCA_000465935	Singleton	Pseudomonas sp. EGD-AK9	65.6	Contig	National Environmental Engineering Research Institute
GCA_000474765	Singleton	Pseudomonas sp. CMAA1215	63.8	Contig	Embrapa
GCA_000582595	Singleton	Pseudomonas sp. BAY1663	65.0	Contig	Bay Zoltan Nonprofit Ltd for Applied Research
GCA_000612585	Singleton	Pseudomonas sp. CHM02	60.9	Contig	Korea Research Institute of Bioscience and Biotechnology
GCA_000632245	Singleton	Pseudomonas sp. RIT357	59.8	Contig	Monash University Malaysia
GCA_000633395	Singleton	Pseudomonas sp. PH1b	62.9	Contig	University of Malaya
GCA_000690905	Singleton	Pseudomonas sp. Ant30-3	58.6	Contig	University of Alabama at Birmingham
GCA_000282975	Singleton	Pseudomonas psychrophila HA-4	56.4	Contig	Harbin Institute of Technology
GCA_000467105	Singleton	Pseudomonas alcaligenes NBRC 14159	64.8	Contig	National Institute of Technology and Evaluation
GCA_000425745	Singleton	Pseudomonas cremoricolorata DSM 17059	63.5	Scaffold	DOE Joint Genome Institute
GCA_000313755	Singleton	Pseudomonas nitroreducens TX1	64.5	Contig	National Central University
GCA_000412695	Singleton	Pseudomonas resinovorans NBRC 106553	65.3	Complete Genome	National Institute of Technology and Evaluation
GCA_000423545	Singleton	Pseudomonas resinovorans DSM 21078	63.6	Scaffold	DOE Joint Genome Institute
GCA_000425625	Singleton	Pseudomonas azotifigens DSM 17556	67.0	Scaffold	DOE Joint Genome Institute
GCA_000425805	Singleton	Pseudomonas vranovensis DSM 16006	61.5	Scaffold	DOE Joint Genome Institute
GCA_000349845	Singleton	Pseudomonas denitrificans ATCC 13867	65.2	Complete Genome	Pusan National University
GCA_000350565	Singleton	Pseudomonas veronii 1YdBTEX2	60.6	Contig	Bacterial Abiotic Stress and Survival Improvement Network - BACSIN
GCA_000410875	Singleton	Pseudomonas pelagia CL-AP6	57.4	Contig	KOPRI
GCA_000621225	Singleton	Pseudomonas mosselii DSM 17497	64.0	Scaffold	DOE Joint Genome Institute
GCA_000498575	Singleton	Pseudomonas taeanensis MS-3	60.9	Contig	Korea Basic Science Institute
GCA_000517305	Singleton	Pseudomonas cichorii JBC1	58.1	Complete Genome	National Academy of Agricultural Science, RDA
GCA_000632535	Singleton	Pseudomonas bauzanensis	61.8	Contig	Tianjin University
GCA_000275905	Singleton	Pseudomonas fluorescens NZI7	63.2	Contig	The Sainsbury Laboratory
GCA_000411615	Singleton	Pseudomonas putida MTCC 5279	62.5	Contig	CSIR-National Botanical Research Institute
GCA_000730425	Singleton	Pseudomonas fluorescens 1	60.1	Complete Genome	Aalborg University
GCA_000730585	Singleton	Pseudomonas japonica NBRC 103040 = DSM 22348	64.1	Contig	National Institute of Technology and Evaluation
GCA_000737245	Singleton	Pseudomonas syringae 3	57.8	Contig	University of Arizona
GCA_000746525	Singleton	Pseudomonas alkylphenolia	60.6	Complete Genome	Seoul National University
GCA_000759445	Singleton	Pseudomonas lutea	60.2	Contig	Kyungpook National University
GCA_000761545	Singleton	Pseudomonas sp. ML96	64.8	Contig	Institute of Microbiology, ASCR
GCA_000800055	Singleton	Pseudomonas sp. 11/12A	59.6	Contig	DOE Joint Genome Institute
GCA_000021045	NA	Azotobacter vinelandii DJ	65.7	Complete Genome	US DOE Joint Genome Institute (JGI-PGF)
GCA_000019225	NA	Cellvibrio japonicus Ueda107	52.0	Complete Genome	J. Craig Venter Institute
